# Multi-region mapping of ligand binding and structural changes in the β-1 adrenergic receptor using carbene footprinting and mass spectrometry

**DOI:** 10.1039/d5sc05107j

**Published:** 2025-10-01

**Authors:** James R. Lloyd, Arppana S. Varughese, Parth Kapoor, Katharina L. Dürr, Hsin-Yung Yen, Ali Jazayeri, Jonathan T. S. Hopper, Neil J. Oldham

**Affiliations:** a School of Chemistry, The University of Nottingham, University Park Nottingham NG7 2RD UK neil.oldham@nottingham.ac.uk; b OMass Therapeutics, Chancellor Court John Smith Drive, ARC Oxford OX4 2GX UK Jonathan.Hopper@omass.com

## Abstract

G protein-coupled receptors (GPCRs) control many physiological processes and are major targets for therapeutic intervention. Transmembrane proteins, such as GPCRs, are inherently flexible and dynamic and often challenging to study using conventional structural methods. Here, we report the use of carbene footprinting to investigate ligand binding and structural changes in the turkey β_1_-adrenergic receptor (*t*β_1_AR), a GPCR and therapeutic target of beta-blocker drugs. The method revealed differences between binding of the agonist, isoprenaline, and the inverse agonist, carazalol, both in terms of their occupancy of the orthosteric ligand binding site and their effects on key regulatory structural features of *t*β_1_AR including the ‘ionic lock’ between transmembrane (TM) helicies 3 and 6. Addition of nanobodies (Nbs) known to stabilise the activated complex (Nb80) and inactivated complex (Nb60) of *t*β_1_AR induced further structural changes above those seen with the ligands alone.

## Abstract 2

We show that photochemical carbene labelling of the turkey β_1_-adrenergic receptor using a generic diazirine probe reveals binding of agonists and antagonists together with resulting conformational changes.

## Graphical abstract

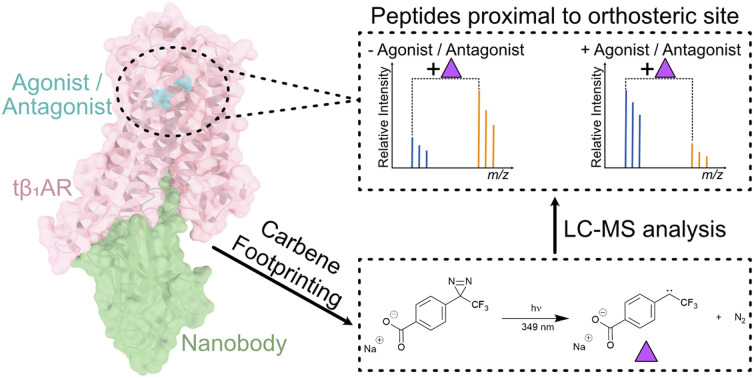

## Introduction

Protein interactions are central to almost every biological process. Understanding these interactions is crucial to elucidate biological systems and advance targeted drug discovery.^1^ X-ray crystallography and cryogenic electron microscopy (cryo-EM) have defined the field of structural biology through atomic level information on protein structure and interactions, but they suffer from relatively low sensitivity and slow turn-around times, with integral membrane proteins (IMPs) providing an extra level of complexity. Mass spectrometry (MS) methods – and more specifically, MS-based chemical labelling techniques – are increasingly used to provide structural data by exploiting the analytical speed and sensitivity afforded by MS.^2^ These approaches incorporate chemical reagents to label accessible residues of a protein, usually in the presence and absence of a binding partner. Interaction with a binding partner shields contact interfaces from chemical modification whilst, in control samples, these sites remain accessible for labelling. Proteolytic digestion and LC-MS analysis permit detection of peptide labelling as well as quantification and comparison of chemical modification between samples. Changes to peptide labelling in the presence of a binding partner may then be used as a reporter for binding interaction sites and dynamics. Ion fragmentation methods can also be employed to identify amino acid (and sub-residue) level chemical labelling changes, enabling structural interrogation of protein complexes. A range of MS-based chemical labelling techniques exist depending on the chemical label employed, including hydrogen–deuterium exchange (HDX), hydroxyl-radical protein footprinting (HRPF), including fast photochemical oxidation of proteins (FPOP), and carbene footprinting.^3–6^

**Scheme 1:**
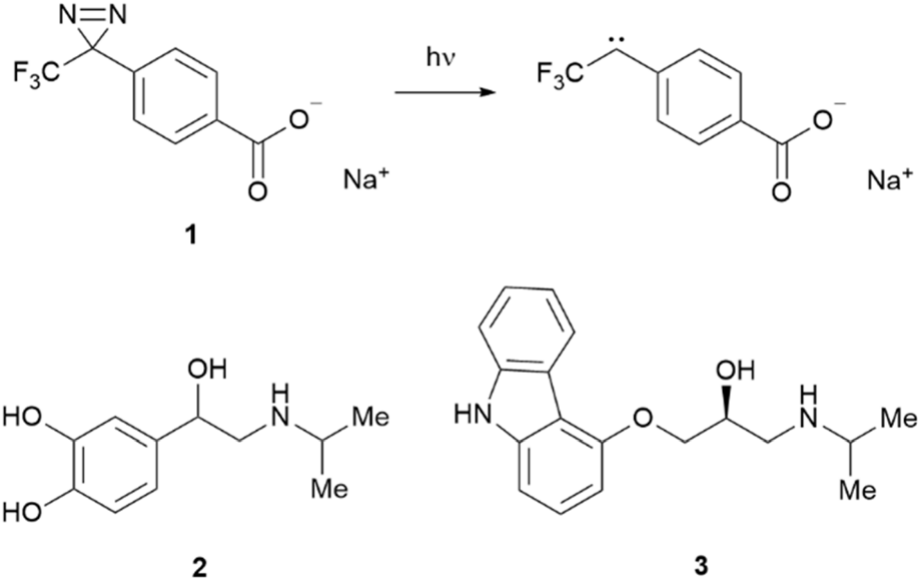
Production of the carbene footprinting label from NaTDB (1) and the structures of the β_1_AR ligands (±)-isoprenaline (2) and (*S*)-carazalol (3).

Carbenes are highly reactive intermediates consisting of a neutral divalent carbon atom with two non-bonding electrons. They are often generated from photolytic activation of diazirines at *ca.* 350 nm. Carbenes undergo a range of insertion and addition reactions, including X–H bond insertion (where X is C, N, O, S) on a ns timescale, leading to mass increases that are easily recognised by MS. Our diazirine of choice is sodium 4-[3-(trifluoromethyl)-3H-diazirin-3-yl]benzoate (NaTDB, 1, Scheme 1). This probe displays high solubility in aqueous solution and efficient incorporation to protein structure upon generation of the carbene, leading to a mass shift of +202 Da. NaTDB has been used to study protein interactions of several clinically relevant systems.^7–10^ Whilst techniques such as HDX, FPOP and LiP-MS have been applied to investigate interactions between membrane proteins and small molecule ligands,^11,12^ carbene footprinting has yet to be utilised for this purpose.

G protein coupled receptors (GPCRs) are the largest family of transmembrane proteins.^13^ They play vital roles in signal transduction by responding to a diverse set of ligands and have been implicated in the perception of smell, taste and light.^14^ Due to their role in vertebrate physiology, GPCRs are frequently implicated in disease. They are also a rich source of therapeutic targets for the pharmaceutical industry.^15^ GPCRs are biochemically unstable, however, and do not lend themselves towards structural interrogation. It has only been relatively recently that the field of GPCR structural biology has emerged owing to breakthroughs in X-ray crystallography, mutationally thermostabilised or fusion proteins and nanobody (Nb) stabilisation. Nbs are the recombinant minimal-sized, antigen-binding domains of heavy chain antibodies, and are uniquely derived from camelids, such as llamas.^16^

GPCRs consist of an extracellular *N*-terminus accompanied by seven transmembrane helices (TMs) that are linked by three extracellular loops (ECLs) and three intracellular loops (ICLs) as well as a final intracellular *C*-terminus. GPCR activity is associated with ligand binding to the extracellular side of the receptor, at the orthosteric site. Binding induces structural rearrangements in TMs, facilitating interaction of G proteins with the receptor's intracellular region.^13^ Bound G proteins can then generate an intracellular signalling response by activating effector enzymes, such as kinases, phospholipases, and adenylyl cyclases.^17^

The βAR-adrenergic receptors (βAR) are members of the Rhodopsin family (R) of GPCRs. They consist of three subtypes: β_1_-adrenergic receptor (β_1_AR), β_2_-adrenergic receptor (β_2_AR) and the β_3_-adrenergic receptor (β_3_AR). β_1_AR is predominantly expressed in cardiac tissue where it plays a key role in heart function. This receptor is endogenously activated by the catecholamine hormone adrenaline, which leads to increased heart rate as part of the familiar fight or flight response.^18^ Medicinally, β_1_AR may be targeted by agonists, such as (±)-isoprenaline (2), for the treatment of bradycardia (abnormally low heart rate),^19^ or inverse agonists, such as (*S*)-carazalol (3) and other beta-blockers, for the treatment of cardiac arrythmia and high blood pressure.^20^ Several structures of β_1_AR bound to agonists, inverse agonists and antagonists have been released.^21–23^ Some of these have included ternary complexes bound to G protein mimics, including nanobodies (Nbs) and more recently, G proteins and β-arrestins.^24–26^ Nbs are being increasingly employed as chaperones to preserve transient protein states and highlight the conformational range of GPCRs.^24^ A range of Nbs with G protein-like properties have been developed that stabilise various activation states of βARs.^27^ Nb80, for example, stabilises β_1_AR in the fully activated state in the presence of an agonist ligand.^23^ Nb60, in contrast, stabilises the inactive state in the presence of an inverse agonist.^27^ Structural comparison between these states has provided valuable insight into the conformational rearrangements associated with β_1_AR activation.

By studying activated and inactivated ligand-turkey β_1_AR-Nb ternary complexes, here we report the first use of carbene footprinting to map the interactions between a membrane protein and its ligands. MS/MS of labelled peptides allowed direct protein-ligand and protein–protein interactions as well as binding partner-induced structural protein changes to be mapped to resolution approaching the amino acid level. These results demonstrate that carbene footprinting can provide rapid and high-quality structural information for this important and challenging class of proteins.

## Results and discussion

### Optimisation of proteolytic conditions

Following successful expression and purification of thermostabilised turkey β_1_AR (*t*β_1_AR), and the nanobodies Nb80 and Nb60 (Fig. S1 and S2), enzymatic digestion and carbene labelling conditions were optimised. This ensured that observed peptides and covalent modification by NaTDB were at suitable levels to report on differential binding partner effects.

*In silico* digestions were performed on the sequences of all proteins. This revealed that chymotrypsin was most suited for digestion of *t*β_1_AR (Fig. S3). In-gel digestion of *t*β_1_AR with chymotrypsin in the presence of ProteaseMAX (an MS-compatible surfactant) generated the highest experimental sequence coverage of all trialled experimental digestion conditions (66%) (Fig. S4). *In silico* digestion of Nb80 and Nb60 showed the greatest predicted sequence coverage with trypsin and chymotrypsin (Fig. S5 and S6). Experimental in-gel digestion of Nb80 and Nb60 showed high sequence coverage with trypsin (Fig. S7 and S8). Screening of diazirine probe concentration revealed that NaTDB at 20 mM provided the greatest carbene modification to *t*β_1_AR (Fig. S9). The carbene labelled chymotryptic peptide coverage was 59% whilst labelled tryptic peptide coverage was 29%. The combined labelled chymotryptic and tryptic digest corresponded to 64% unique sequence coverage of the receptor. This was lower than ideal, but the presence of large hydrophobic transmembrane peptides rendered further coverage by LC-MS challenging, despite repeated efforts. Most importantly, good coverage of the ligand binding pocket and intracellular G-protein interacting region was obtained. For labelled coverage of the Nbs, NaTDB at 20 mM was also found to be optimal (Fig. S10 and S11). Labelled Nb80 tryptic peptide coverage was 90% and labelled Nb60 tryptic peptide coverage was 88%.

### Differential study of Turkey beta-1 adrenergic receptor

After optimising *t*β_1_AR sequence coverage and labelling conditions, we sought to capture interaction and conformational changes of *t*β_1_AR in the presence of orthosteric ligands and intracellular domain binding partners using carbene footprinting.

Enzymatic digestion of *t*β_1_AR was carried out separately with chymotrypsin and trypsin to maximise peptide coverage and the associated insights into receptor interactions and dynamics. Carbene labelling of peptides, expressed as fractional modification (*f*_mod_) of each peptide, was compared between unbound and ligand-treated *t*β_1_AR, using either isoprenaline or carazalol, and also between ligand-treated *t*β_1_AR and ternary receptor complexes, which included the activating or deactivating Nb, respectively. MS/MS was performed on selected peptides which displayed carbene labelling changes. Binding of both small molecule ligands and Nbs to *t*β_1_AR were required to saturate the activated or inactivated receptor state. Based on crystal structures, binding of ligands alone was not expected to induce conformational changes in the receptor, and masking was only anticipated at the extracellular orthosteric binding site.^23^

#### Isoprenaline (2) binding

**Fig. 1:**
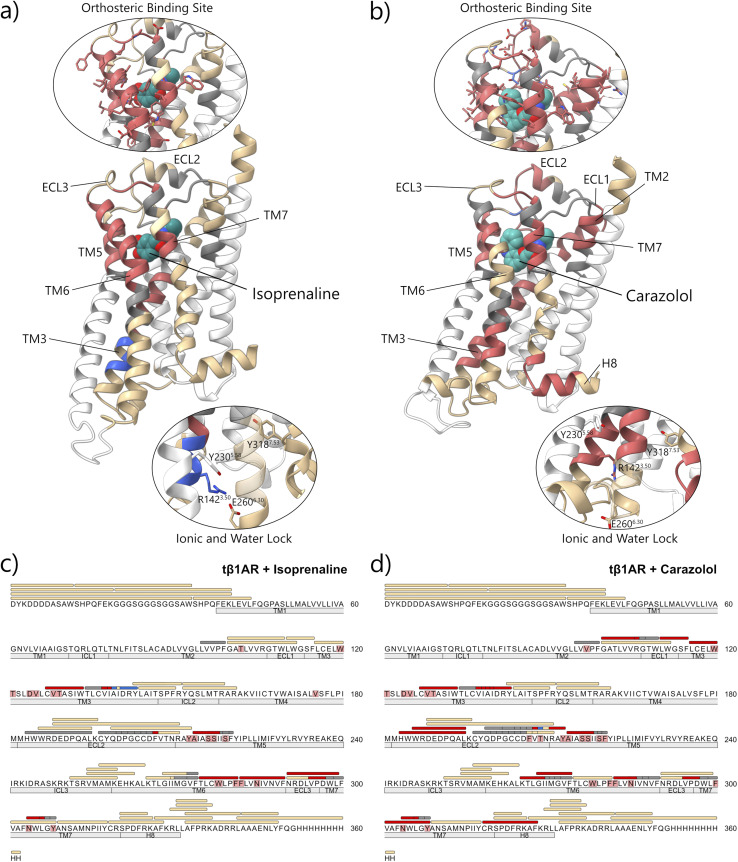
Carbene footprinting of *t*β_1_AR in the absence and presence of either agonist isoprenaline (a and c) or antagonist carazolol (b and d). (a and b) Observed carbene labelling coverage of *t*β_1_AR mapped onto the crystal structure. Close-ups of the regions covering the orthosteric binding site and the water and ionic lock are also shown. (c and d) Sequence map showing the sequence and labelling coverage of *t*β_1_AR. Colours indicate significant changes in labelling upon ligand binding: red = significant decrease in labelling in the presence of the ligand; blue = significant increase in labelling in the presence of the ligand; tan = no difference in labelling; grey = no labelling coverage; white = no sequence coverage. Regions corresponding to transmembrane helices, intracellular and extracellular loops are shown below the sequence. *t*β_1_AR residues highlighted in red indicate regions predicted to interact with the respective ligand. (Significance determined by Student's *t*-test; *p* ≤ 0.05; *n* = 4). Active isoprenaline-bound structure adapted from PDB: 2Y03.^32^ Inactive carazolol-bound structure adapted from PDB: 5JQH.^27^

Reductions in carbene modification, as measured by the relative change in *f*_mod_ compared to unbound *t*β_1_AR, were observed on several *t*β_1_AR peptides in the presence of the agonist isoprenaline, including chymotryptic peptides 127^3.35^–133^3.41^, 211^5.39^–219^5.47^, 275^6.45^–281^6.51^, 282^6.52^–290^6.60^, 291^6.61^–299^7.34^, 291^6.61^–300^7.35^ & 304^7.39^–308^7.43^, and tryptic peptide 195–208^5.36^ (residue numbering is based on the employed construct sequence; however, the Ballesteros–Weinstein numbering scheme is also indicated in superscript where available^28^), see Fig. 1a, c, S12 and S13. When mapped to the *t*β_1_AR structure, masking effects localised to the extracellular binding cavity, and highlighted capture of membrane protein-ligand interactions using carbene footprinting for the first time (Fig. 1a).

Interestingly, MS/MS of peptide 134^3.42^–143^3.51^ showed that Val-137 and Ile-138 were masked, whilst Ala-139, Asp-141, Arg-142 and Tyr-143 were unmasked by isoprenaline binding. In the inactive state of *t*β_1_AR, Arg-142 on TM3 is known to form an ‘ionic lock’ with Glu-260 on TM6. The breaking of this lock by binding of the activating ligand provides a rationale for the observed differences in labelling around Arg-142 (Fig. 1a).

**Fig. 2:**
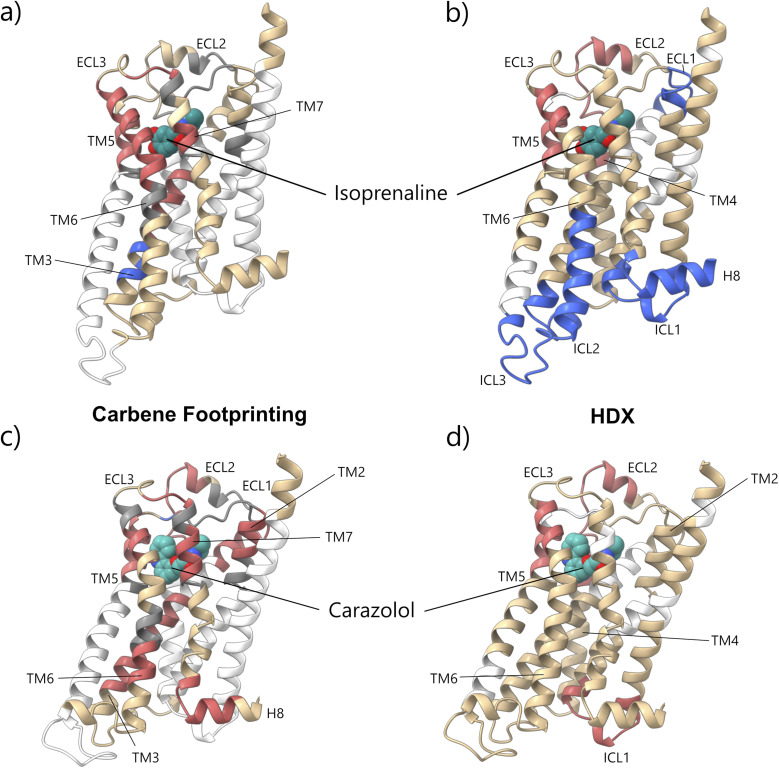
Comparison of differential carbene footprinting *vs.* HDX (Toporowska *et al.*, 2025)^29^ of *t*β1AR in the presence and absence of (a and b) isoprenaline and (c and d) carazolol. Significant increases in either fractional modification (a and c) or deuterium uptake (b and d) are highlighted in blue, whilst significant decreases in either fractional modification (a and c) or deuterium uptake (b and d) are highlighted in red. No changes are highlighted in tan and no coverage is represented by white. No carbene labelling coverage is represented in grey. Active isoprenaline-bound structure adapted from PDB: 2Y03.^32^ Inactive carazolol-bound structure adapted from PDB: 5JQH.^27^

Recently, Toporowska *et al.* performed an HDX study of *t*β_1_AR that also showed protection of the orthosteric site due to isoprenaline binding.^29^ However, in addition, they reported deprotection in the intracellular loops and proximal helices upon binding of this ligand. With the exception of ICL2, a combination of either low sequence coverage or 100% labelling prevented us from reporting effect in this region (see Fig. 2a and b).

#### Carazalol (3) binding

For carazalol-treated *t*β_1_AR, as well as those peptides listed above for isoprenaline, additional masking was observed on peptides 101^2.61^–110^23.50^, 111^23.51^–115^3.23^, 116^3.24^–120^3.28^, 183^4.64^–193, 185–195, 197–210^5.38^ & 301^7.36^–308^7.43^, located on the extracellular tip of TM2, 3 and 4 (Fig. 1d, S14 and S15). When mapped onto the structure of *t*β_1_AR these changes were found to be proximal to the orthosteric binding pocket (Fig. 1b), due to the larger size of carazalol, leading to additional contacts with the protein. This finding demonstrated the ability of carbene labelling to differentiate between ligand size, and associated occupancy of the *t*β_1_AR binding pocket: a feature which could be of considerable utility in understanding structure–activity relationship (SAR) within medicinal chemistry campaigns. Carazalol-induced masking was also observed on the intracellular tip of TM3/ICL2 and TM6 (including peptides: 265^6.35^–274^6.44^, 268^6.38^–274^6.44^ & 319^7.54^–328^8.54^) which suggested distal protection effects caused by binding of the inverse agonist. In general, there was consistency between differential labelling of tryptic and chymotryptic peptides except in a small number of cases where an *F*_mod_ of 1.0 was seen in both the presence and absence of ligand.

The masking of peptides located in proximity to the orthosteric site was consistent with findings from the HDX study conducted by Toporowska *et al.* (see Fig. 2c and d).^29^ They observed protection of three peptides in this region, overlapping with the masked regions seen in this study for both ligands. However, they were not able to observe the additional masking proximal to the orthosteric site or the distal masking unique to the carazolol-only condition.

Our results suggested further effects on protein structure away from the binding site despite unliganded and carazolol-bound crystal structures being highly similar and displaying an RMSD of 0.737 Å. Interestingly, this was in agreement with observations made by West and colleagues from HDX experiments on the related β_2_AR receptor, where very similar effects were seen on reduction of deuterium uptake for analogous regions of the receptor in the presence of carazolol.^30^ Thus, it may be that ‘static’ crystal X-ray data are not reporting all the structural effects of carazalol binding that occur in the detergent-solubilised state. Moreover, in the HDX study, this reduction in exchange was not seen with isoprenaline, which is also in agreement with our findings.

As with isoprenaline binding, peptide 134^3.42^–143^3.51^ showed interesting behaviour in the presence of carazalol. This time, however, residues Val-137 to Tyr-143 all displayed masking, unlike with isoprenaline, where some residues were masked and some unmasked (see above and Fig. 1b). This suggested stabilisation of the ionic lock by the inverse agonist, a result that was consistent with deactivation of the receptor. This was an especially notable result as, when combined with the isoprenaline experiments (see above), changes in labelling of this peptide were able to distinguish between *t*β_1_AR activation and deactivation.

#### Isoprenaline (2) and Nb80 binding

*t*β_1_AR was next treated with Nb80, in the presence of isoprenaline, mimicking the fully activated G-protein bound ternary complex, and the peptide *f*_mod_ compared to untreated receptor. The addition of Nb80 and ligand showed small increased masking effects of three peptides at the orthosteric site when compared with isoprenaline alone, specifically peptides: 127^3.35^–133^3.41^, 291^6.61^–299^7.34^ & 304^7.39^–308^7.43^ (Fig. 2a, c, S16 and S17). Additionally, several masking effects on ICL2/TM3, containing the ionic lock, were identified in the active ternary receptor complex with isoprenaline and Nb80 (including chymotryptic peptides 134^3.42^–143^3.51^ and 144^3.52^–150^34.51^ and tryptic peptide 143^3.51^–151^34.52^). The observed decreases in labelling in this region are indicative of Nb80 binding, and/or conformational changes associated with receptor activation, especially as multiple residues covering this region are within 5 Å of Nb80. This proximity to the nanobody may have also prevented the capture of the disruption of the ionic lock upon activation seen in the isoprenaline-only condition.

Interestingly, once again, our results are in agreement with the HDX study conducted on *t*β_1_AR by Toporowska and colleagues.^29^ In the presence of a combination of isoprenaline and miniGs, protection of a *t*β_1_AR peptide covering residues 140^3.48^–150^34.51^ was observed, but no significant changes in deuterium uptake in the isoprenaline-alone condition were seen, indicative of protection being a result of miniGs binding.

With the addition of Nb80, labelling reductions on the central portion of *t*β_1_AR's TM6 were also observed, specifically on overlapping peptides 265^6.35^–274^6.44^ and 268^6.38^–274^6.44^. Movement of TM6 away from the helical bundle is a quintessential molecular switch of GPCR activation but it remained unknown how such a conformational change would affect chemical accessibility to the labelling probe and carbene modification of the helix. Interrogation of activated ternary structures highlighted Nb80 binding to ICL2/TM3/7 and the observed masking effects likely reflected protein–protein contacts. Chymotryptic peptide 257^6.27^–264^6.34^ and tryptic peptide 252^6.22^–259^6.29^ on TM6 displayed 100% carbene labelling in the presence or absence of the Nbs which impeded our ability to detect binding events in this region of the receptor. These peptides span the junction between TM6 and ICL3, and were expected to be accessible in both activated and deactivated states in spite of the conformational change. However, use of a trypsin digestion allowed us to discern an unmasking event on the intracellular tip of TM6 (peptide 255^6.25^–262^6.32^) in the ternary complex compared to isoprenaline-only complex (Fig. S13 and S17). This likely reflected the classical movement of TM6 upon receptor activation since a gain in carbene modification would not be expected to accompany direct protein–protein binding site events.

#### Carazalol (3) and Nb60 binding

**Fig. 3:**
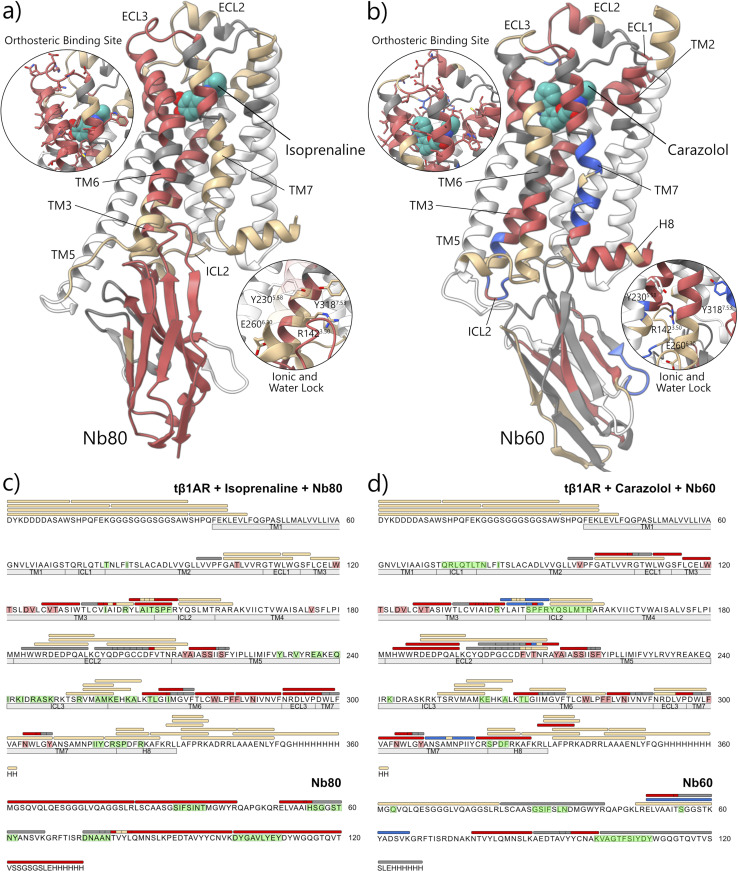
Carbene footprinting of *t*β_1_AR in the absence and presence of either agonist isoprenaline and Nb60 (a and c) or antagonist carazolol and Nb80 (b and d). (a and b) Observed sequence and labelling coverage of *t*β1AR-Nb complex with bound ligand (cyan) mapped onto the crystal structure. Close-ups of the regions covering the orthosteric binding site and the water and ionic lock are also shown. (c and d) Sequence map showing the sequence and labelling coverage of the *t*β_1_AR-Nb complex with bound ligand. Colours indicate significant changes in labelling compared to unbound state: red = significant decrease in labelling; blue = significant increase in labelling; tan = no difference in labelling; grey = no labelling coverage; white = no sequence coverage. Regions corresponding to transmembrane helices, intracellular and extracellular loops are shown below the sequence. *t*β_1_AR residues highlighted in red indicate regions predicted to interact with the respective ligand. *t*β_1_AR and Nb80 residues highlighted in green represent regions within 5 Å of each other. (Significance determined by Student's *t*-test; *p* ≤ 0.05; *n* = 4). Active isoprenaline and Nb80-bound structure adapted from PDB: 6H7J.^23^ Inactive carazolol and Nb60-bound structure adapted from PDB: 5JQH.^27^

In the inactive ternary complex of carazolol-*t*β_1_AR-Nb60, labelling experiments revealed no additional masking around the orthosteric site than already seen with carazolol alone (Fig. 3b, d and S18). As with the carazolol-only condition, peptide 134^3.42^–143^3.51^ (covering the ionic lock) displayed masking, again indicating stabilisation of the inactive state of *t*β_1_AR by the intact salt bridge. However, additional unmasking was seen of the chymotryptic peptide (144^3.52^–150^34.51^) adjacent to the ionic lock compared to carazolol-*t*β_1_AR alone, indicative of appreciable conformational change resulting from Nb60 binding. Unmasking was also seen of peptide 309^7.43^–318^7.53^ located on the TM7 region and MS/MS analysis localised this unmasking to two regions: between residues Ala-309 to Ala-312 and between residue Asn-314 and Tyr-318. The activated form of *t*β_1_AR is known to be stabilised by the formation of a hydrogen bond between Tyr-318 and Tyr-230 on TM5 coordinated by a water molecule, referred to as the water lock (Fig. 3b). The unmasking seen therefore implies that the deactivation of the structure led to disruption of this water lock and that Nb60 is required to stabilise the fully deactivated state of *t*β_1_AR. These findings also suggest that unliganded *t*β_1_AR may have a partially activated structure or intact water lock even in the absence of ligand-binding.

Tryptic peptide 143^3.51^–151^34.52^ also highlighted unmasking on TM3/ICL2, supporting the chymotrypsin data above (Fig. S19). Additionally, tryptic peptide 330^8.56^–336, part of the short H8 showed masking with the introduction of Nb60. Chymotryptic peptides in this region were 100% labelled, and thus the tryptic peptide provided additional information. The observed masking may have indicated interaction with the Nb60, or some additional stabilisation caused by its binding. Proximity to the *C*-terminal His-tag, which is absent from published structures, made accurate comparison of this effect difficult. The *t*β_1_AR-Nb60 contact surface primarily comprised of TM3/5/6 and our analysis of these helices was largely limited by poor peptide sequence coverage and high carbene modification.

#### Nb masking

Carbene footprinting was also applied to Nbs in the presence of *t*β_1_AR. Nb80 treated with *t*β_1_AR displayed extensive reductions in carbene modification across every tryptic peptide, compared with control samples (Fig. S20). This was explained by the long tryptic peptides which were generated, where each peptide spanned the length of the protein and presumably contained residues involved in binding to *t*β_1_AR, or that were at least proximal to it. Indeed, analysis of the crystal structure revealed that every tryptic Nb80 peptide that we observed contained at least one residue that was within 6 Å of *t*β_1_AR. MS/MS analysis was impeded by low levels of labelling across the masked peptides however sub-peptide labelling analysis of two peptides was conducted.

Fragmentation of labelled peptide 49–67 (ELVAAIHSGGSTNYANSVK) showed that all carbene modification (and masking effects) were located on residues 49–53 (ELVAA), Ile54 and His55. Examination of the crystal structure (Fig. 3a) revealed that these residues were located within the intracellular binding cavity of *t*β_1_AR which again indicated binding of Nb80 to *t*β_1_AR. Similarly, fragmentation of labelled peptide 75–101 (DNAANTVYLQMNSLKPEDTAVYYCNVK) showed that masking effects were located on Thr80, Leu83 and residues 84–101 (QMNSLKPEDTAVYYCNVK). Although these residues were located distally from *t*β_1_AR, the *C*-terminal end of peptide 84–101 was located proximal to the interaction site and it appeared plausible that the masking effect on this peptide reflected this contact interface. Nb80 treated with *t*β_1_AR and isoprenaline also displayed reductions in carbene modification across all tryptic peptides, compared with control samples, in agreement with previous observations (Fig. S21). This again highlighted binding of the Nb to *t*β_1_AR, irrespective of agonist binding which was in accordance with the literature. Recently Qiu *et al.* have recently examined the masking of miniGs by β_1_AR in the presence and absence of isoprenaline using HDX. Here they found decreased deuterium uptake on helix 5 of the miniGs in the presence of isoprenaline, demonstrating the effect of the ligand on this interaction.^31^

Nb60 displayed three masking effects and one unmasking event in the presence of *t*β_1_AR compared to without *t*β_1_AR (Fig. S22). Reductions in labelling were observed on peptides 48–60 (ELVAAITSGGSTK), 78–88 (NTVYLQMNSLK) and 89–100 (AEDTAVYYCNAK) whilst the gain in fractional modification was seen on peptide 48–66 (ELVAAITSGGSTKYADSVK). These more conservative labelling changes in the presence of the receptor (compared to with Nb80) reflected the difference in TM6 conformation where the helix encapsulated less of the Nb60. Indeed, 19 residues on Nb60 were within 5 Å of *t*β_1_AR compared to 27 residues on Nb80. Masking on peptide 48–60 reinforced Nb interaction with *t*β_1_AR. MS/MS revealed specific masking effects on residues 48–53 (ELVAAI) and Thr54 (Fig. S22b and c), which were located on and around the CDR2 loop. This region is known to form contacts with *t*β_1_AR and the observed masking effects presumably reflect reduced chemical accessibility of the probe. An unmasking effect was located on the missed cleavage peptide 48–66. Given that we had conducted sub-peptide labelling analysis of peptide 48–60, we were able to conclude that the unmasking event occurred on the subsequent residues 61–66 (YADSVK). Examination of the crystal structure revealed that these residues were located on a loop, distal from *t*β_1_AR, implying a gain in chemical accessibility and labelling due to a probable conformational change associated with *t*β_1_AR–Nb binding. Masking of peptide 89–100 also reiterated Nb60-*t*β_1_AR binding. This peptide was located on β-strand B, adjacent to the CDR2 loop. Pleasingly, MS/MS analysis revealed that carbene modification was located towards the *C*-terminal side of the peptide, specifically on residues 96–100 (YCNAK), which were proximal to the *t*β_1_AR-Nb60 contact interface. These results again highlighted interaction between Nb60 and *t*β_1_AR.

The tryptic digest of Nb60 in the presence of *t*β_1_AR and carazolol compared to without *t*β_1_AR showed identical labelling changes to Nb60 treated with the receptor alone (Fig. S23). This was anticipated since binding of Nbs to βARs is not dependent on small molecules, and consistent with what was observed for Nb80. The HDX study by Qiu *et al.* found no change in deuterium uptake on helix 5 of the miniGs in the presence of carazalol.^31^

## Conclusions

In summary, we have applied carbene footprinting mass spectrometry to the study of a GPCR for the first time. The technique was performed on agonist and inverse agonist-bound turkey β_1_AR as well as fully active and inactive ternary receptor-Nb complexes. The ability to use flexible protease combinations with carbene labelling workflows, in this case trypsin and chymotrypsin, allow the peptide sequence coverage of the receptor to be maximised. In each case, carbene footprinting was able to accurately map the *t*β_1_AR orthosteric binding site and enable characterisation of ligand binding. Extended masking was observed around the binding cavity in carazolol-treated samples compared to isoprenaline-treated samples which may have reflected the inverse agonist's bulkier size and expanded contact network. Changes in carbene labelling were also observed on the intracellular side of the receptor in ternary *t*β_1_AR-Nb complexes. These differed between isoprenaline and carazolol-treated ternary complexes, reflecting capture of Nb binding and highlighting the conformational range between active and inactive states. Carbene footprinting was also performed on Nbs in the presence of *t*β_1_AR and either ligand. Large changes in fractional modification were identified over the surface of either Nb, further reiterating binding of the heavy-chain antibody fragments to the receptor.

This work demonstrates the feasibility of using carbene footprinting and mass spectrometry to understand and characterise membrane protein (and in particular, GPCR)-ligand interactions and induced conformational changes well as identifying druggable pockets.

## Author contributions

The project was conceived by N. J. O., J. T. S. H. and A. J., investigation was conducted by J. R. L., A. S. V., P. K., K. L. D. and H. Y. Y., formal data analysis was performed by J. R. L. and A. S. V., supervision was provided by N. J. O. and J. T. S. H., and the manuscript was written by J. R. L., A. S. V., N. J. O. with review and editing by J. T. S. H.

## Conflicts of interest

There are no conflicts to declare.

## Supplementary Material

SC-OLF-D5SC05107J-s001

## Data Availability

Data in support of this work, together with experimental methods, are freely available in the supplementary information (SI) associated with this publication. Unprocessed mass spectrometry raw data files can be found in the University of Nottingham public data repository at DOI https://www.doi.org/10.17639/nott.7534. Table S7 lists the files available and a brief description of the corresponding experiment. See DOI: https://doi.org/10.1039/d5sc05107j.
