# Supplementary material for: Multi-region mapping of ligand binding and structural changes in the β-1 adrenergic receptor using carbene footprinting and mass spectrometry
Source: Chem Sci. 2025 Oct 1;16(43):20527–35. doi: 10.1039/d5sc05107j (PMC12501881; doi:10.1039/d5sc05107j)
Supplement: SC-016-D5SC05107J-s001 [file SC-016-D5SC05107J-s001.pdf]

Supplementary Information (SI) for Chemical Science.
This journal is © The Royal Society of Chemistry 2025

Multi-region mapping of ligand binding and structural changes in the β-1 adrenergic receptor using carbene footprinting and mass spectrometry

James R. Lloyd,[a] Arppana S. Varughese,[a] Parth Kapoor,[b†] Katharina L. Dürr,[b] Hsin-Yung Yen,[b] Ali Jazayeri,[b,‡] Jonathan T.S. Hopper*[b] and Neil J. Oldham*[a]

[a]School of Chemistry, University of Nottingham, University Park, Nottingham NG7 2RD, U.K.

[b]OMass Therapeutics, Schrodinger Building, Oxford Science Park, Oxford OX4 4GE, U.K.

[†] Current Address: UCB Pharma, 216 Bath Road, Slough, Berkshire, SL1 3WE, UK.

[‡] SV Health Investors. 71 Kingsway, London, WC2B 6ST, UK

*Authors for correspondence

Neil Oldham neil.oldham@nottingham.ac.uk

Jonathan Hopper jonathan.hopper@omass.com

Methods

Protein production

β_1AR. Sf9 cells (Invitrogen) overexpressed with the thermostabilised *Meleagris gallopavo* (turkey) beta-1 adrenergic receptor (β_1AR) construct[1] (GenScript) with an N-terminal FLAG and strep tag and C-terminal His-tag using the Bac-to-Bac baculovirus expression system (Thermo Fisher Scientific) were provided by OMass Therapeutics, as described in Toporowska et al., 2024. The cell pellet was defrosted on ice and resuspended with lysis buffer (1 mM EDTA, 20 mM Tris pH 8, 15 mL) and transferred to a 500 mL measuring beaker. Cell lysis buffer was added to the pellet to bring the final volume to 300 mL. The resuspension was passed twice through a M-110 PS microfluidizer (Microfluidics) at 8,000 psi. This was collected and transferred to ultracentrifuge tubes (Beckman Coulter) and centrifuged at 9000 g for 20 min in an Optima XPN-80 Ultracentrifuge (Beckman Coulter) using a Type 45 Ti rotor (Beckman Coulter) for 20 min at 4 °C to pellet cell debris. The supernatant was collected and transferred to 65 mL ultracentrifuge tubes (Beckman Coulter). Samples were centrifuged at 170 000 g in an Optima XPN-80 Ultracentrifuge (Beckman Coulter) using a Type 45 Ti (Beckman Coulter) Rotor for 2 h at 4 °C to pellet cell membranes. Membranes were transferred to a borosilicate dounce homogeniser (Thermo Fisher Scientific) and manually homogenised in resuspension buffer (1 mM EDTA, 20 mM Tris pH 8, 40 mL). The resuspension was made up to a final volume of 300 mL with resuspension buffer and transferred to 65 mL ultracentrifuge tubes (Beckman Coulter) before centrifuging at 170 000 g in an Optima XPN-80 Ultracentrifuge (Beckman Coulter) using a Type 45 Ti (Beckman Coulter) rotor for 2 h at 4 °C. This process was repeated twice using wash buffer (0.2 mM EDTA, 20 mM Tris pH 8) and then membrane resuspension buffer (20 mM Tris pH 8) with the latter being resuspended to a final volume of 30 mL in 15 mL Falcon tubes (Thermo Fisher Scientific). These were flash frozen in liquid nitrogen and stored at −80 °C overnight.

Membranes were defrosted on ice and incubated at an equal volume of solubilisation buffer (700 mM NaCl, 6 mM Imidazole, 3 % DDM, 20 mM Tris pH 8) before vortexing for a few seconds. Solubilised membranes were centrifuged at 120 000 g in an Optima XPN-80 Ultracentrifuge (Beckman Coulter) using a Type 45 Ti (Beckman Coulter) Rotor for 2 h at 4 °C to pellet any remaining unsolubilised cell membranes. The supernatant was collected and diluted 1.5-fold with dilution buffer (350 mM NaCl, 3 mM Imidazole, 20 mM Tris pH 8). The solution was passed through a Millex-GV syringe filter (0.45 µm, 33 mm, Merck). A 5 mL HiTRAP TALON column (Cytiva) was installed on an AKTA Pure (Cytiva) that had been equilibrated in buffer A (350 mM NaCl, 3 mM Imidazole, 0.05 % DDM, 20 mM Tris pH 8) at a flow rate of 2 mL/min. The filtered supernatant was loaded onto the column at a flow rate of 0.5 mL/min and the flow-through collected. The column was washed with 100 % buffer B (350 mM NaCl, 250 mM Imidazole, 0.05 % DDM, 20 mM Tris pH 8) for 10 column volumes and the eluent was also collected. Fractions were run on a 12 % SDS-PAGE gel and eluent corresponding to β_1AR was concentrated to a final volume of 7 mL using a protein concentrator (100 kDa, Thermo Fisher Scientific). A HiTRAP 5 mL Sephadex G-25 desalting column (Cytiva) was installed on an AKTA Pure (Cytiva) that had been equilibrated in buffer A at a flow rate of 2 mL/min. Concentrated β_1AR was injected onto a 2 mL loop and eluted in buffer A at a flow rate of 1 mL/min in a 96-well plate. Fractions corresponding to β_1AR were collected, concentrated (0.75 mg/mL) and stored at −80 °C.

Nanobodies. Genes encoding Nb80 and Nb60 were cloned into pET22b(+) expression vectors containing an N-terminal pelB leader sequence and C-terminal His-tag (GenScript). Nb expression plasmid was transformed into BL21 cells (Agilent). A single colony was inoculated in LB (50 mL) supplemented with carbenicillin (50 µg/mL) and incubated at 220 rpm and 37 °C for 18 h. Cultures were diluted in LB (OD_{600} 0.1, 1 L) supplemented with carbenicillin (50 µg/mL). Cultures were incubated

at 220 rpm and 37 °C until an OD_{600} value of 0.6 was reached using a DS-11 FX 42 Spectrophotometer/Fluorometer (DeNovix). At this point cultures were removed from the incubator for 30 min whilst the incubator temperature was lowered to 18 °C. Cultures were induced with IPTG (1 mM) and returned to the incubator for 18 h.

Cells were centrifuged at 6000 g in 1 L polypropylene bottles (Beckman Coulter) using an Avanti JXN-26 high-speed centrifuge (Beckman Coulter) containing a JLA-8.1000 rotor (Beckman Coulter) for 10 min at 4 °C. The supernatant was removed, and the cells were resuspended in ice cold TES buffer (20 mM EDTA, 25 mM Tris pH 8, 20 % sucrose (w/v)) supplemented with two cOMPLETE protease inhibitor tablets (Merck). These were incubated at 4 °C with stirring for 1 h. TES/4 buffer (5 mM EDTA, 5 mM Tris pH 8, 5 % sucrose (w/v)) was added to the resuspended cells before stirring on ice for 1 h. The cell lysate was centrifuged at 10 000 g for 30 min at 4 °C. The supernatant was transferred to clean tubes and MgCl2 (2 mM) was added to quench free EDTA. Cell lysate was incubated with Talon resin (1 mL) for 1 h at 4 °C. The supernatant was added to a 2.5 cm x 30 cm glass Econo-Column (BioRad) and the flow-through collected. Immobilised protein was washed with 20 CV wash buffer (20 mM HEPES pH 7.5, 200 mM NaCl, 5 mM Imidazole). Protein was eluted with elution buffer (20 mM HEPES pH 7.5, 200 mM NaCl, 150 mM Imidazole). Protein was buffer exchanged into wash buffer, concentrated and stored at −80 °C.

Carbene labelling

Photochemical labelling of β_1AR. Aqueous isoprenaline (1 mM, 2 µL) was added to two buffered (350 mM NaCl, 3 mM Imidazole, 0.05 % DDM, 20 mM Tris pH 8) solutions of β_1AR (18 µM, 15 µL). MQQ water (2 µL) was added to another buffered solution of β_1AR (18 µM, 15 µL). All three samples were incubated on ice for 5 min. Buffered (20 mM HEPES pH 7.5, 200 mM NaCl, 5 mM Imidazole) Nb80 (173 µM, 2 µL) was added to an isoprenaline-treated β_1AR solution. Nb80 buffer was added to the remaining two β_1AR solutions. Samples were incubated for a further 35 min. An aqueous solution of the NaTDB (100 mM, 5 µL) was combined with ligand-treated and control β_1AR samples and incubated for 10 min. Aliquots (5 µL) were transferred to tapered autosampler vials (four replicates) and flash-frozen with liquid nitrogen. Samples were irradiated for 15 s using a Explorer One 349 laser (actively Q-switched Nd:YLF laser 349 nm wavelength, 1000 Hz repetition frequency, 125 µJ pulsed energy, Spectra Physics) that was vertically refracted into the vials by a 45° mirror.

Carazolol in DMSO (1 mM, 2 µL) was added to two buffered solutions of β_1AR (18 µM, 15 µL). DMSO (10 %, 2 µL) was added to another buffered solution of β_1AR (18 µM, 15 µL). All three samples were incubated on ice for 5 min. Nb60 (186 µM, 2 µL) was added to a carazolol-treated β_1AR solution. Nb60 buffer was added to the remaining two β_1AR solutions. Samples were incubated for a further 35 min. An aqueous solution of the NaTDB (100 mM, 5 µL) was combined with ligand-treated and control β_1AR samples and incubated for 10 min. Aliquots (5 µL) were transferred to tapered autosampler vials (four replicates) snap-frozen and irradiated at 349 nm as described above.

Photochemical labelling of Nanobodies. β_1AR (18 µM, 50 µL) was added to a protein concentrator (100K MWCO, 0.1-0.5 mL, Thermo Fisher Scientific) that had been equilibrated with β_1AR buffer A (Table X). This was centrifuged at 3000 g until the sample volume had at least halved.

Aqueous isoprenaline (1 mM, 2 µL) was added to a buffered solution of β_1AR (36 µM, 16 µL). MQ water (2 µL) was added to a separate buffered solution of β_1AR (36 µM, 16 µL). β_1AR samples were

incubated on ice for 5 min. Both β_1AR samples were separately added to Nb80 (186 µM, 2 µL). MQ water (2 µL) and β_1AR buffer (16 µL) were also added to a separate Nb80 sample (186 µM, 2 µL). All three samples were incubated for a further 35 min. An aqueous solution of the NaTDB (100 mM, 5 µL) was combined with ligand-treated and control Nb80 samples and incubated for 10 min. Aliquots (5 µL) were transferred to tapered autosampler vials (four replicates), snap-frozen and irradiated at 349 nm as described above.

Carazolol in DMSO (1 mM, 2 µL) was added to a buffered solution of β_1AR (36 µM, 16 µL). DMSO (10 %, 2 µL) was added to a separate buffered solution of β_1AR (36 µM, 16 µL). β_1AR samples were incubated on ice for 5 min. Both β_1AR samples were separately added to Nb60 (173 µM, 2 µL). DMSO (10 %, 2 µL) and β_1AR buffer (16 µL) were also added to a separate Nb60 sample (173 µM, 2 µL). All three samples were incubated for a further 35 min. An aqueous solution of the NaTDB (100 mM, 5 µL) was combined with ligand-treated and control Nb60 samples and incubated for 10 min. 50 Photochemical labelling Aliquots (5 µL) were transferred to tapered autosampler vials (four replicates), snap-frozen and irradiated at 349 nm as described above.

Sample preparation and analysis

Sodium dodecyl sulfate–polyacrylamide gel electrophoresis. Protein samples were combined with 6X SDS-PAGE reducing buffer (375 mM Tris-HCl pH 8.0, 9 % (w/v) SDS, 50 % (v/v) glycerol, 0.03 % (w/v) bromophenol blue, 9 % (v/v) βME, 1 µL) and incubated at room temperature for 10 min. These were loaded onto a 12 % TGX SDS-PAGE gel (BioRad). SDS-PAGE was conducted at 160 V for 50 min (PowerPac Basic, BioRad). The gel was washed with MQ water and heated by microwaving on 'high' for 2 min. The water was discarded, and the process repeated twice more. The gel was then microwaved on medium with SimplyBlue SafeStain (Thermo Fisher Scientific) for 90 s and then further incubated at room temperature on a rocker for 10 min. The stain was removed, and the gel was washed with MQ water and heated by microwaving on 'medium power' for 2 min. The water was discarded, and the process repeated twice more.

In-gel proteolytic digestion. Protein bands were excised using a scalpel, cut into 1 mm^2 pieces and destained with aqueous acetonitrile (MeCN, 50 %, 50 µL) for 10 min at room temperature. Gel pieces were dehydrated with MeCN (450 µL) with agitation for 3 min before the MeCN supernatant was removed. Gel pieces were treated with DTT solution to reduce protein disulfide bonds (10 mM, Merck, ammonium bicarbonate (AmBic) 100 mM, 50 µL) at 55 °C for 30 min before being dehydrated with MeCN (450 µL), and the supernatant removed. Gel pieces were then treated with iodoacetamide solution (55 mM, Merck, AmBic 100 mM, 50 µL) and incubated in the dark for 30 min to alkylate cysteine thiol groups before again being dehydrated with MeCN (450 µL), with removal of the supernatant. Gel pieces were finally incubated with protease solution (10 ng/µL, AmBic 50 mM, 50 µL) at the relevant temperature for 18 hour (chymotrypsin – 25°C, trypsin – 37°C and pepsin – 37°C). Formic acid (10 µL) was added to protein digests and kept on ice. Supernatant was removed from the gel pieces and centrifuged at 5000 g for 3 min. This was transferred to plastic autosampler vials for nano-LC-MS analysis.

Liquid chromatography-mass spectrometry. Digests were analysed with a Dionex U3000 nano-LC coupled to a ThermoFisher LTQ FT Ultra Mass Spectrometer (Thermo Fisher Scientific) containing a nano-ESI source. An injection volume of 3 µL was loaded onto a C18 Pepmap300 loading column (10 mm, 300 Å, 5 µm particle size, Thermo Fisher Scientific). Sample separation was performed using a C18 Pepmap300 column (150 mm × 75 µm, 300 Å, 5 µm particle size, Thermo Fisher Scientific) with a

flow rate of 400 nL min^{-1} and a gradient of two mobile phases: mobile phase A (5 % MeCN, 0.1 % formic acid) and mobile phase B (95 % MeCN, 0.1 % formic acid) run as follows: t = 0min 100% A, t = 30 min 45% A, t = 30.5 min 10% A, t = 35min 10% A, t = 35.5 min 100% A, t = 50 min 100% A.

The mass spectrometer was operated in positive ion mode with uncoated tips (MSWil) in combination with an external voltage supply. The inlet capillary of the mass spectrometer was held at 275 °C with a tube lens value of 145 V. For peptide identification in protease optimisation experiments, a DDA scan mode was employed in which the three most intense ions from each survey scan were fragmented in an LTQ XL LIT and subsequently analysed. Ions were selected within a mass window of 2 Th. A dynamic exclusion list was employed to prevent multiple isolation of the same ion - the repeat count was set at 3 with a window duration of 45 s and an exclusion duration of 360 s. For labelled peptides, a full scan mode was employed. For sub-peptide analysis of carbene modified peptides, CID MS/MS was conducted on selected labelled peptides and detergent. This was carried out with a nominal energy of 35.0 and a mass window of 8 Th. The activation time was set at 30 ms and an activation-Q value of 0.250.

Data analysis

Peptide identification. Peptide identity was confirmed by CID MS/MS of precursor peptide ions and database searching. RAW files were uploaded to SearchGUI version 4.1.1197 and searched against a custom protein sequence database including common contaminants, proteases and the decoy sequence of the protein of interest using the X!Tandem algorithm and results visualised in PeptideShaker version 2.2.8.99[2,3]. Search settings included the relevant protease, a peptide length of 4-40 residues, three missed cleavages, a precursor charge of between +1-5, fragment b and y ions, fixed carbamidomethylation of cysteine residues, a precursor error of 0.05 Da, a fragment ion error of 0.1 Da and an FDR of 1%.

Peptide-level label quantification. Quantification of carbene labelling at the peptide-level was carried out using PepFoot version 1.2.1[4]. RAW files were uploaded onto the software and converted to mz5 filetype. Unlabelled and labelled EICs (of the same peptide) were generated automatically by the software by searching for a 202.02 Da mass shift corresponding to the addition of the carbene probe (on any amino acid) from the unmodified peptide masses (chosen at the peptide identification stage). The spectra were then manually inspected to ensure sampling of the correct ion. This was carried out with a mass tolerance of 0.02 Da, three missed cleavages, a peptide length of 4-40 residues and the relevant protease. Peak areas were integrated, and fractional modifications generated from the ratio of labelled peak area to the sum of the labelled and unlabelled peak areas (Equation 2.1). Fractional modifications for each peptide were compared between treatments. Significant peptide-level labelling differences were discerned by way of a 2-tailed student T-test ($n = 4$; $p \leq 0.05$). Experience has shown that small but statistically significant changes in F_{mod} reveal structurally meaningful changes without co-reference to fold-change values.

$$F_{mod} = \frac{A_{labelled}}{A_{labelled} + A_{unlabelled}} \tag{2.1}$$

Residue-level label quantification. Quantification of carbene labelling at the residue-level was carried out using targeted CID MS/MS of labelled peptides showing a significant change in labelling at the peptide level. MS/MS spectra were combined over retention times pertaining to a particular precursor ion EICs. This was again carried out with a precursor mass tolerance of 0.02 Da. MS/MS spectra were

inspected manually for b and y fragment ions before the ratio of labelled fragment intensities to the sum of labelled and unlabelled fragment intensities of one series were used to calculate residue-level fractional modifications (Equation 2.2). Absolute modifications were determined by calculating the difference in fractional modification between two consecutive fragments and multiplying by the peptide level fractional modification (Equation 2.3). In the event that fragment ions could not be detected, the residue was grouped together with the previous residue(s). Per-residue modifications were averaged across identified fragments and compared between treatments. Significant sub-peptide level labelling differences were discerned by way of a 2-tailed student T-test ($n = 4$; $p \leq 0.05$).

$$F_{mod(n_i)} = \frac{I(n_{i_{labelled}})}{I(n_{i_{labelled}}) + A(n_{i_{unlabelled}})}$$

$$Abs_{mod} = F_{mod}\left[F_{mod(n_i)} - F_{mod(n_i-1)}\right] \quad (2.3)$$

Computational analysis

Homology modelling. The β_1AR-isoprenaline complex was generated by I-TASSER using PDB 2Y03[5] (turkey β_1AR bound to isoprenaline) as a template[6]. The fully activated β_1AR structure was generated by I-TASSER using PDB 6H7J[7] (activated-state of turkey β_1AR bound to isoprenaline and Nb80) as a template[6]. Isoprenaline and Nb80 were aligned to this model with the ChimeraX matchmaker tool using the same PDB template[6]. The fully inactivated β_1AR structure was generated by I-TASSER using PDB was generated using PDB 5JQH[8] (inactivated-state of β_2AR bound to carazolol and Nb60) as a template. Carazolol and Nb60 were aligned to this model with the ChimeraX matchmaker tool using the same PDB template.

Molecular visualisation. Protein structures were visualised using ChimeraX version 1.3.103[9].

In-silico digestion. In-silico digestions were performed using the Peptide Cutter server, utilising three missed cleavages and a peptide length of between 4-40 residues[10].

References

1 Y. Lee, T. Warne, R. Nehmé, S. Pandey, H. Dwivedi-Agnihotri, M. Chaturvedi, P. C. Edwards, J. García-Nafría, A. G. W. Leslie, A. K. Shukla and C. G. Tate, *Nature*, 2020, **583**, 862–866.

2 H. Barsnes and M. Vaudel, *J Proteome Res*, 2018, **17**, 2552–2555.

3 M. Vaudel, J. M. Burkhart, R. P. Zahedi, E. Oveland, F. S. Berven, A. Sickmann, L. Martens and H. Barsnes, *Nat Biotechnol*, 2015, **33**, 22–24.

4 J. Bellamy-Carter and N. J. Oldham, *J Proteome Res*, 2019, **18**, 2925–2930.

5 T. Warne, R. Moukhametzianov, J. G. Baker, R. Nehmé, P. C. Edwards, A. G. W. Leslie, G. F. X. Schertler and C. G. Tate, *Nature*, 2011, **469**, 241–244.

6 J. Yang and Y. Zhang, *Nucleic Acids Res*, 2015, **43**, W174–W181.

7 T. Warne, P. C. Edwards, A. S. Doré, A. G. W. Leslie and C. G. Tate, *Science (1979)*, 2019, **364**, 775–778.

8 D. P. Staus, R. T. Strachan, A. Manglik, B. Pani, A. W. Kahsai, T. H. Kim, L. M. Wingler, S. Ahn, A. Chatterjee, A. Masoudi, A. C. Kruse, E. Pardon, J. Steyaert, W. I. Weis, R. S. Prosser, B. K. Kobilka, T. Costa and R. J. Lefkowitz, *Nature*, 2016, **535**, 448–452.

9 E. F. Pettersen, T. D. Goddard, C. C. Huang, E. C. Meng, G. S. Couch, T. I. Croll, J. H. Morris and T. E. Ferrin, *Protein Science*, 2021, **30**, 70–82.

10 E. Gasteiger, C. Hoogland, A. Gattiker, S. Duvaud, M. R. Wilkins, R. D. Appel and A. Bairoch, in *The Proteomics Protocols Handbook*, Humana Press, Totowa, NJ, 2005, pp. 571–607.

Results

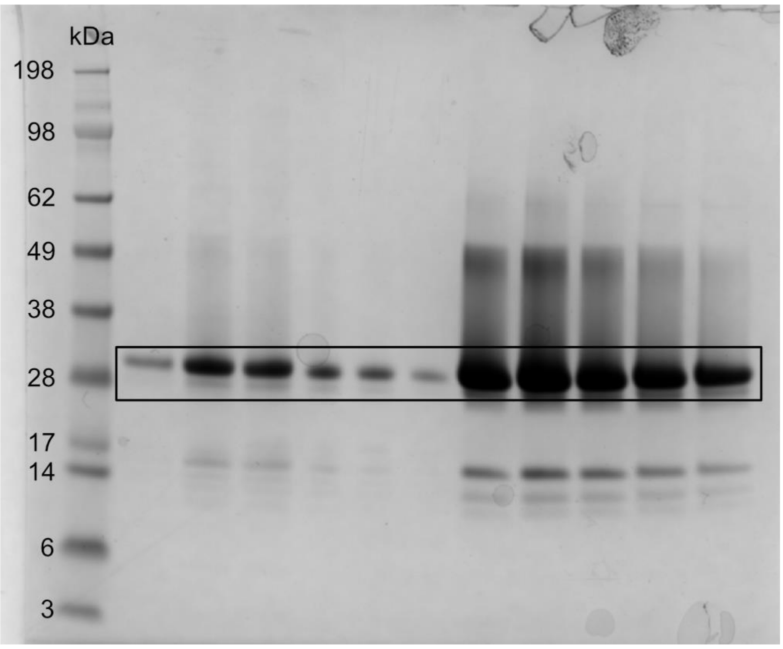

Fig. S1. Purification of DDM solubilised β_1AR. SDS PAGE gel showing factions collected from a HiTRAP TALON IMAC column. Each lane corresponds to a 5 mL fraction collected between elution volumes 205 – 260 mL. The band corresponding to β_1AR is indicated by the black box.

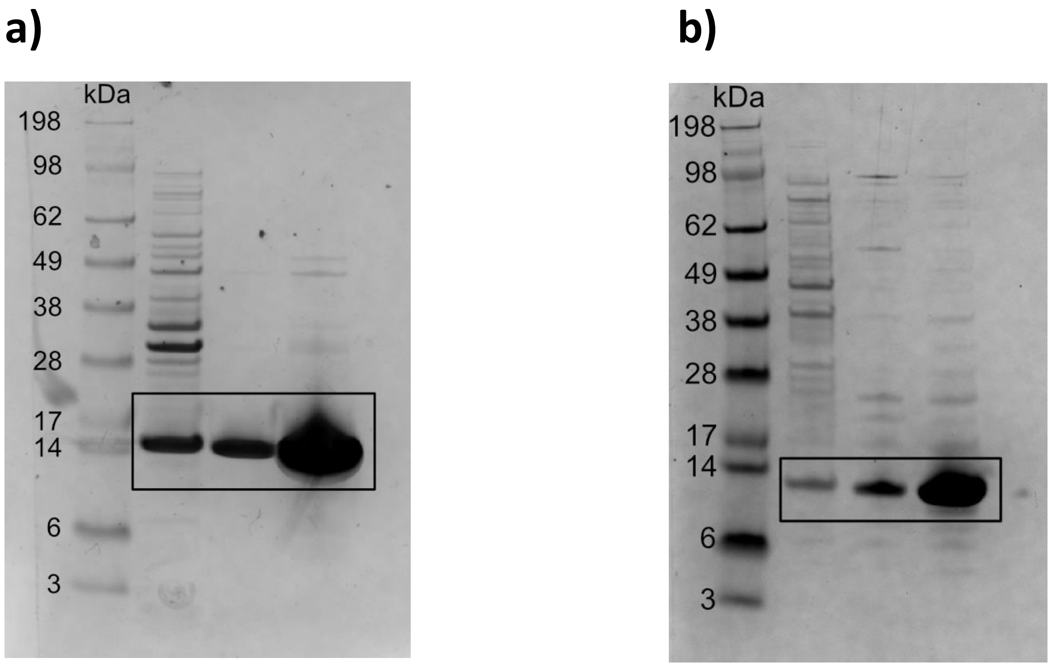

Fig. S2. Purification of nanobodies Nb80 and Nb60. SDS-PAGE gels for (a) Nb80 and (b) Nb60. In each case, lanes correspond to the molecular weight ladder, flow-through, wash and eluent, respectively. Each nanobody is indicated by the black box.

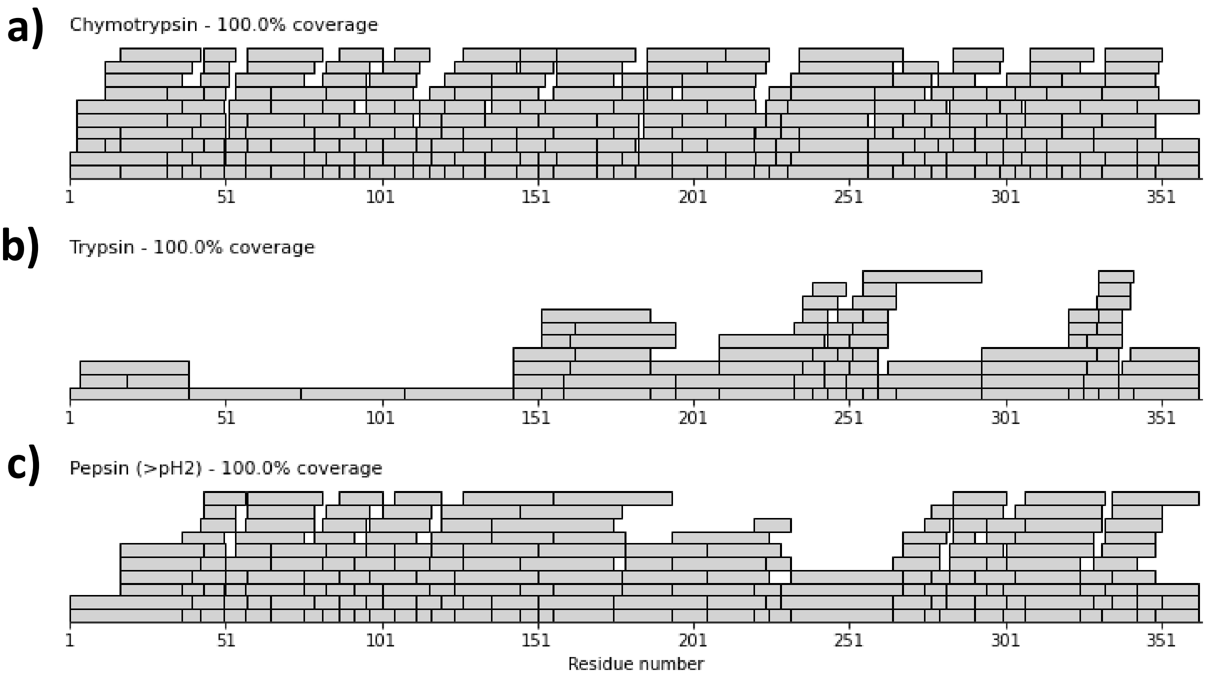

Fig. S3. In-silico digest of β1AR. Theoretical peptide coverage of β1AR with (a) chymotrypsin, (b) trypsin and (c) pepsin using the PeptideCutter Server. Three missed cleavages were employed, and peptides filtered to between 4-40 residues in length.

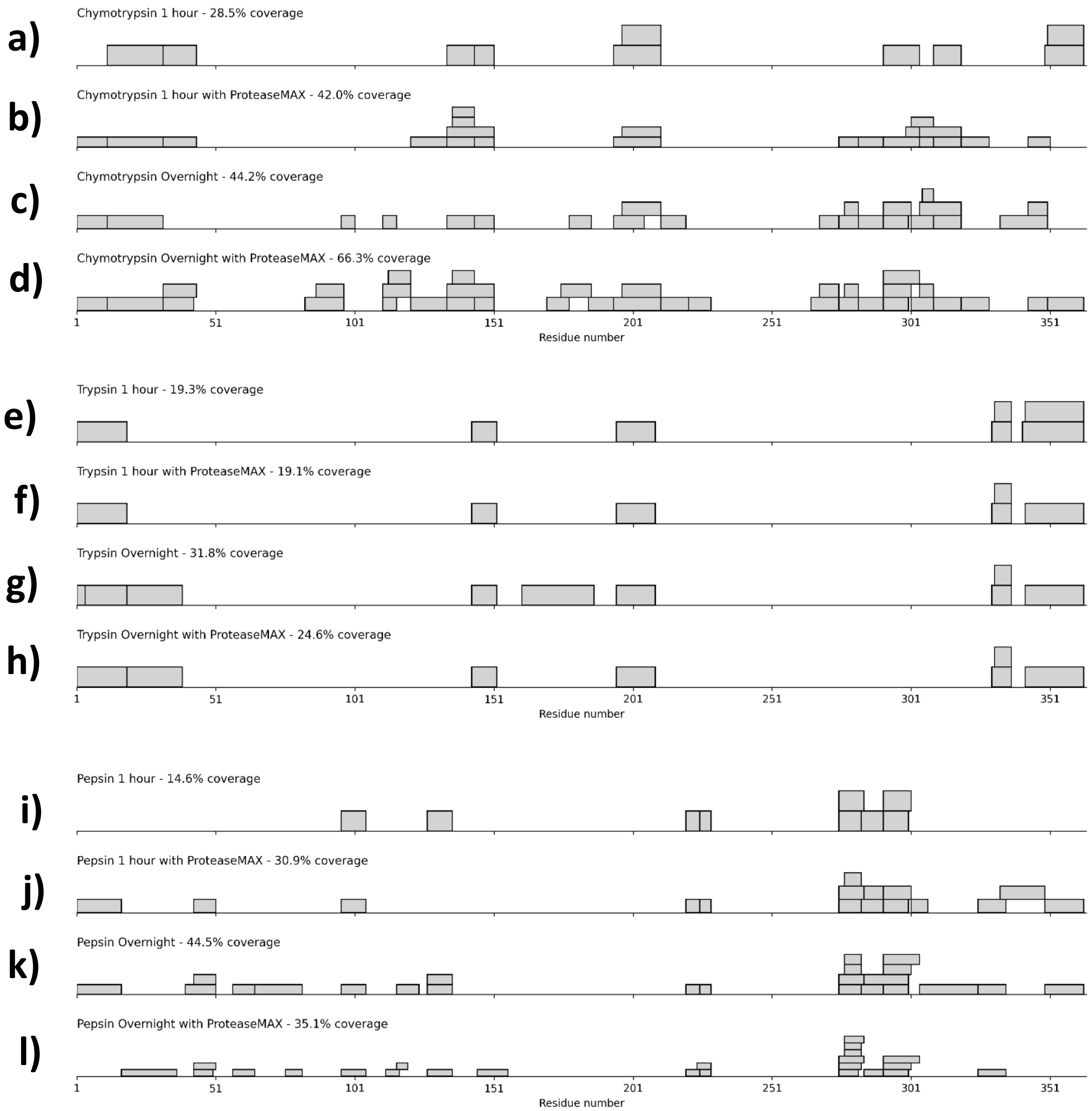

Fig. S4. Experimental digest of β1AR. Observed sequence coverage by DDA LC-MS/MS following chymotrypsin digestion for (a) 1 hr, (b) 1 hr with ProteaseMAX, (c) overnight, (d) overnight with ProteaseMAX, trypsin digestion for (e) 1 hr, (f) 1 hr with ProteaseMAX, (g) overnight, (h) overnight with ProteaseMAX, pepsin digestion for (i) 1 hr, (j) 1 hr with ProteaseMAX, (k) overnight, (l) overnight with ProteaseMAX.

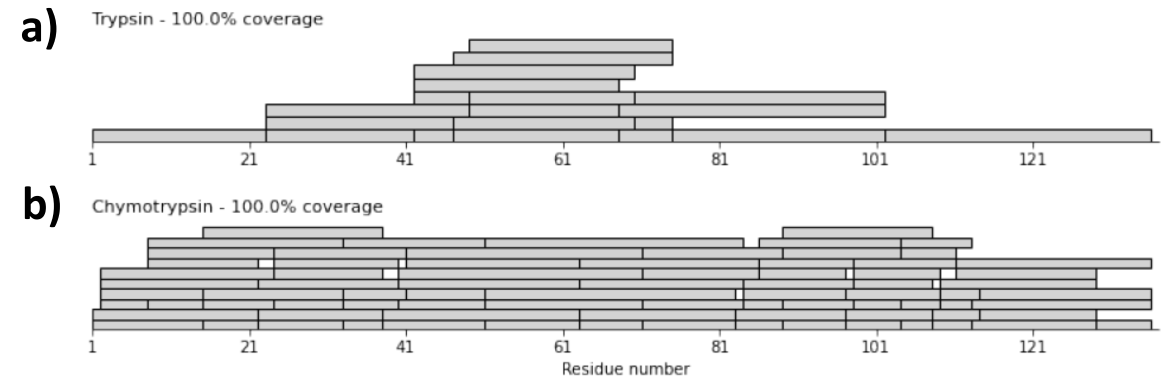

Fig. S5. In-silico digest of Nb80. Theoretical peptide coverage of Nb80 with (a) trypsin and (b) chymotrypsin using the PeptideCutter Server. Three missed cleavages were employed, and peptides filtered to between 4-40 residues in length.

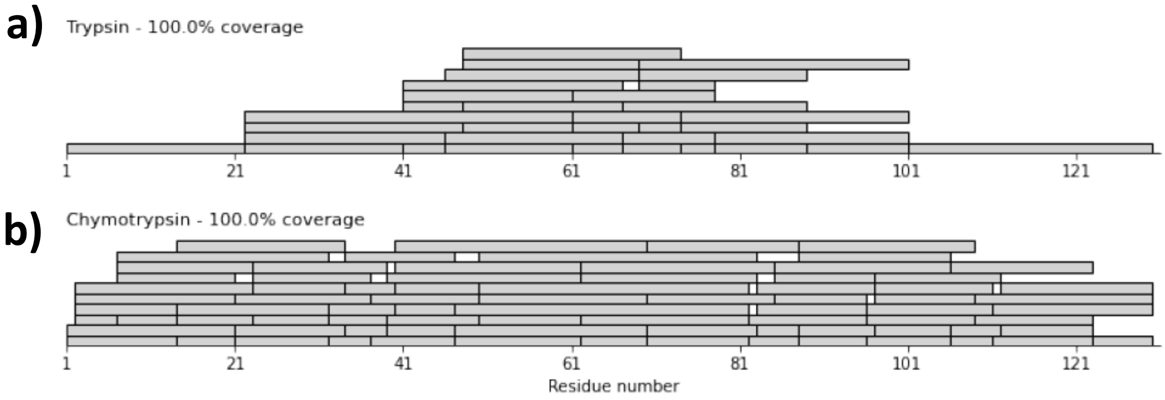

Fig. S6. In-silico digest of Nb60. Theoretical peptide coverage of Nb60 with (a) trypsin and (b) chymotrypsin using the PeptideCutter Server. Three missed cleavages were employed, and peptides filtered to between 4-40 residues in length.

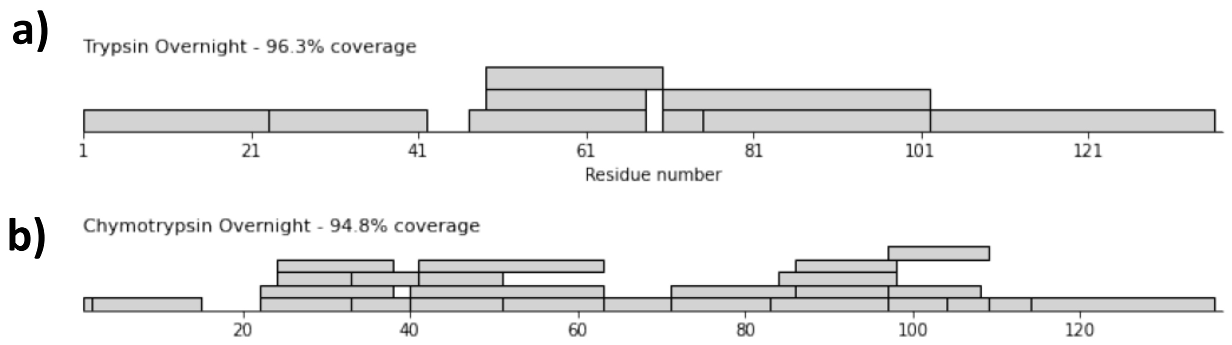

Fig. S7. Experimental digest of Nb80. Observed sequence coverage by DDA LC-MS/MS following (a) trypsin digestion overnight and (b) chymotrypsin digestion overnight.

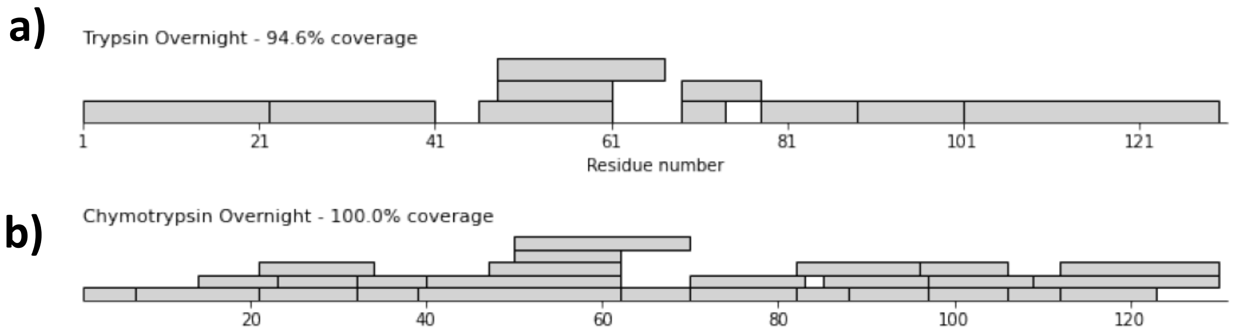

Fig. S8. Experimental digest of Nb60. Observed sequence coverage by DDA LC-MS/MS following (a) trypsin digestion overnight and (b) chymotrypsin digestion overnight.

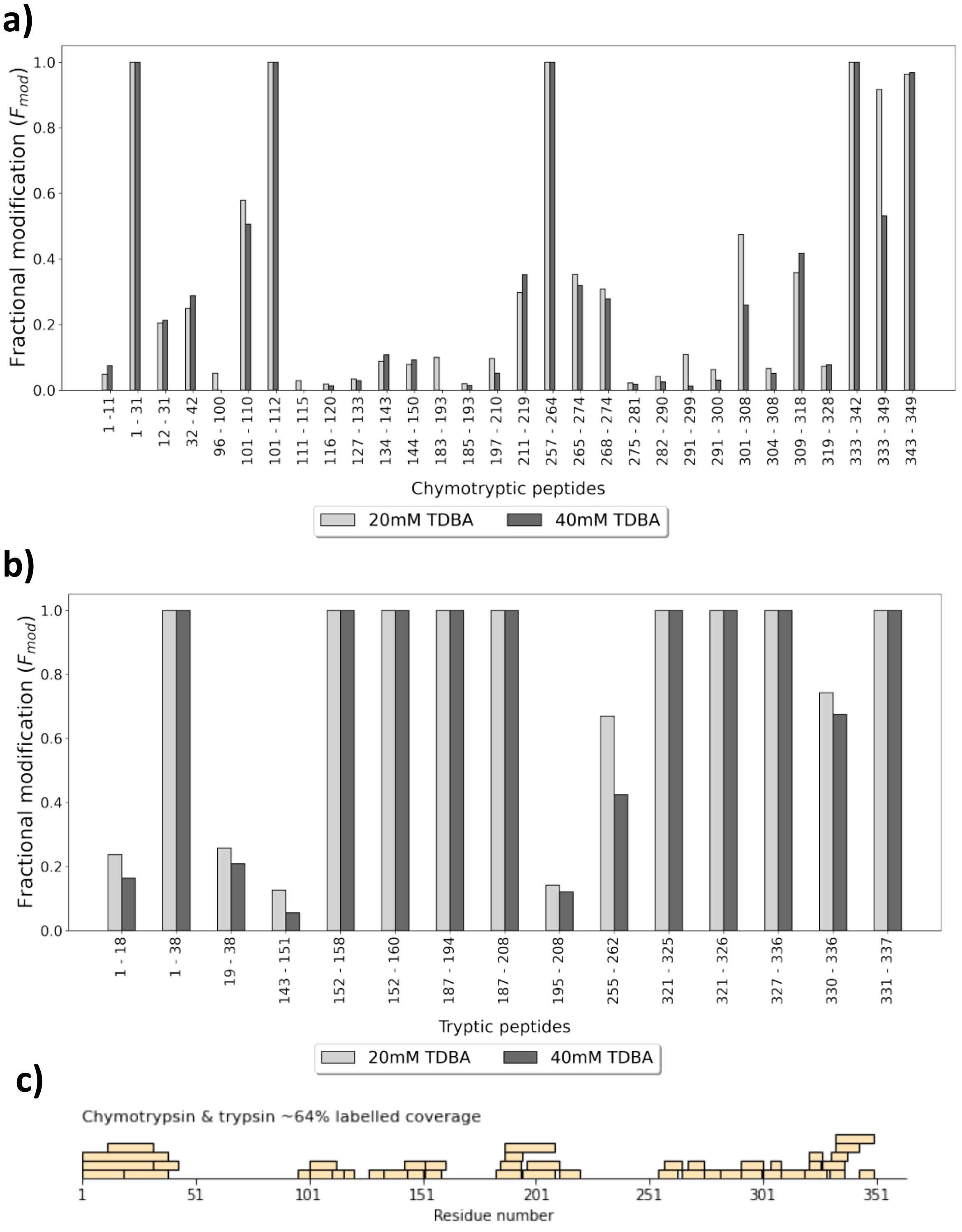

Fig. S9. Carbene labelling coverage of β1AR. Labelled peptides observed with (a) chymotrypsin and (b) trypsin at 20 mM and 40 mM TDBA sodium salt. (c) combined labelled peptide coverage (chymotrypsin and trypsin) at 20 mM TDBA sodium salt.

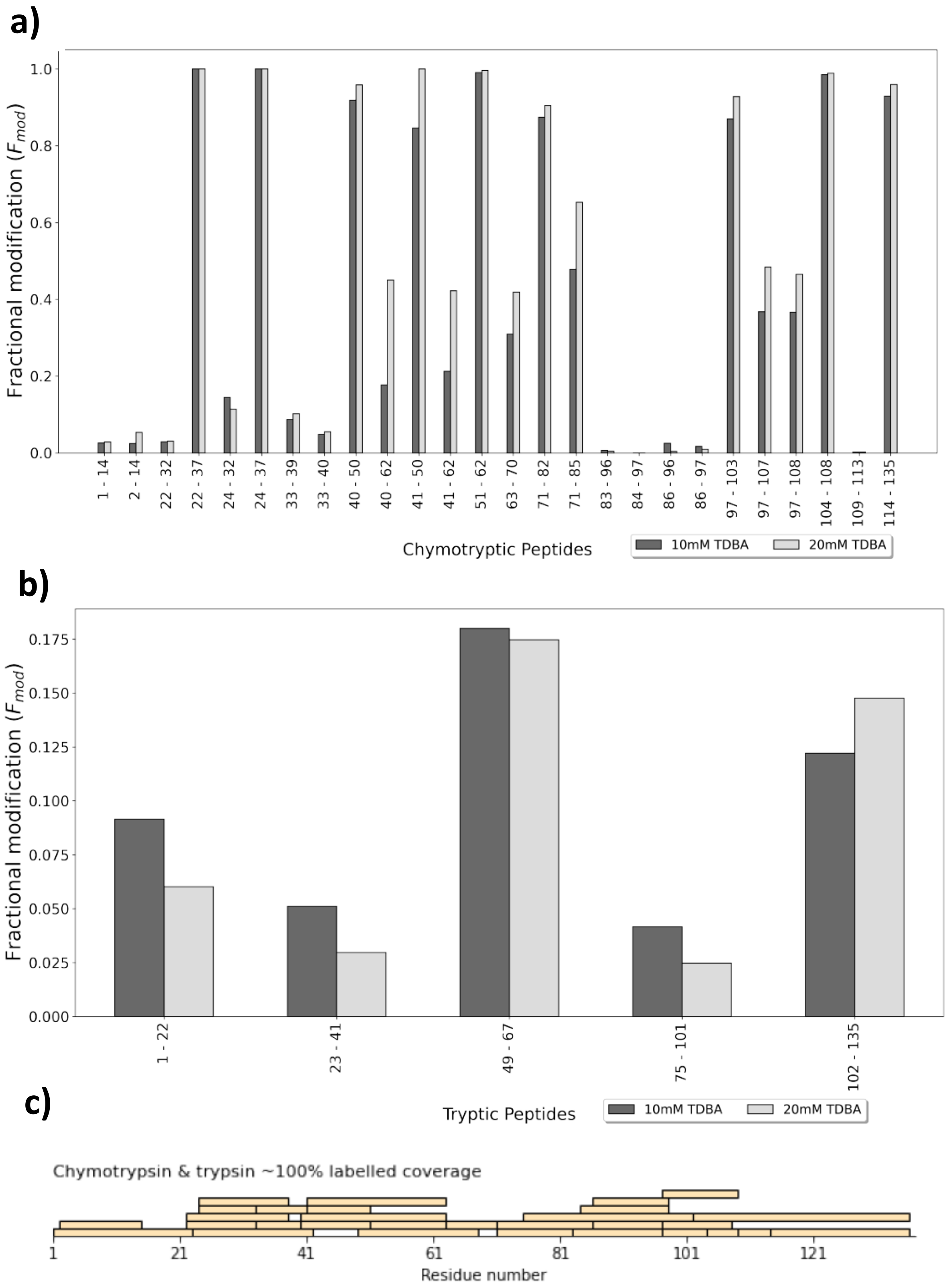

Fig. S10. Carbene labelling coverage of Nb80. Labelled peptides observed with (a) chymotrypsin and (b) trypsin at 10 mM and 20 mM TDBA sodium salt. (c) combined labelled peptide coverage (chymotrypsin and trypsin) at 20 mM TDBA sodium salt.

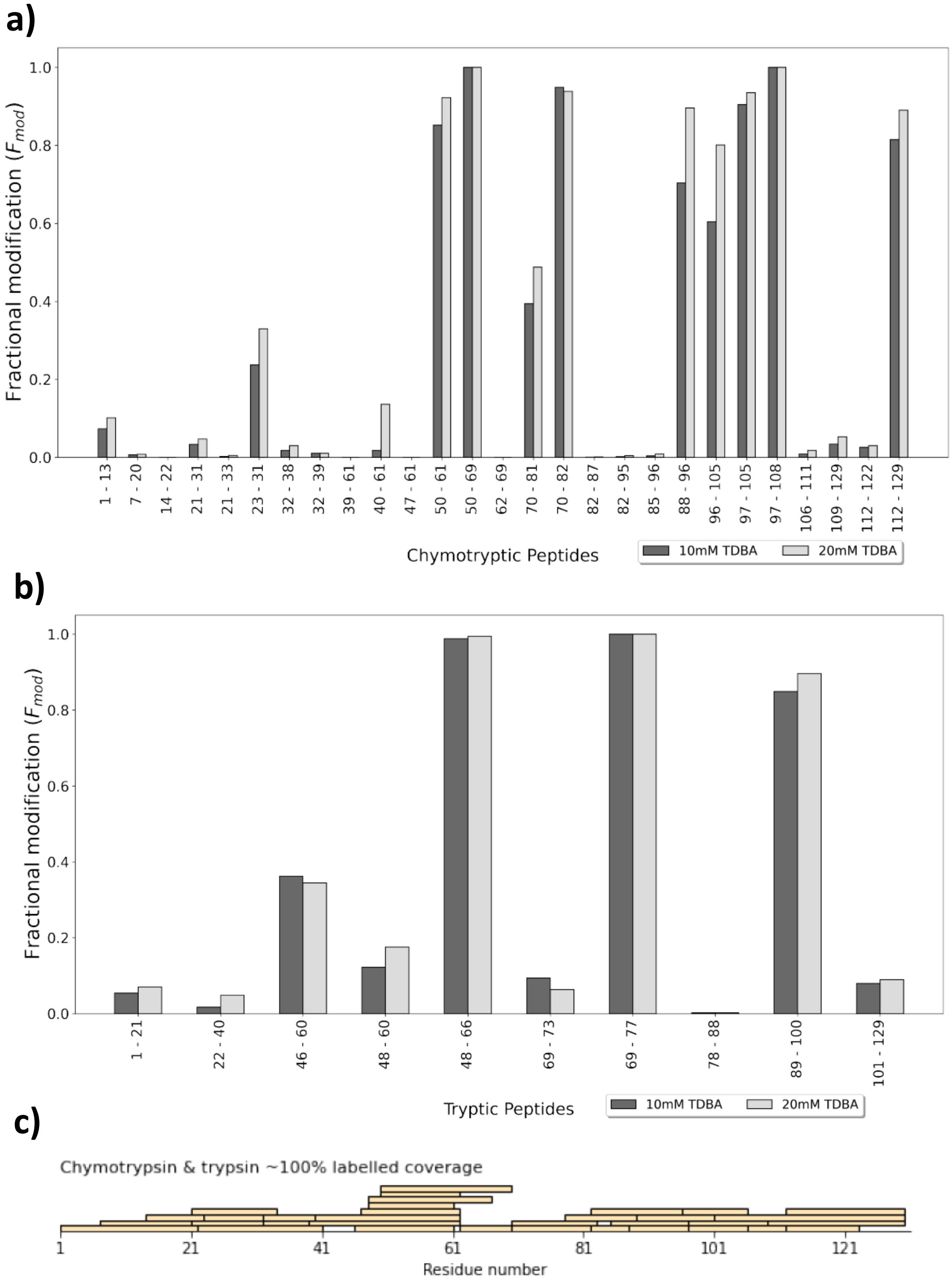

Fig. S11. Carbene labelling coverage of Nb60. Labelled peptides observed with (a) chymotrypsin and (b) trypsin at 10 mM and 20 mM TDBA sodium salt. (c) combined labelled peptide coverage (chymotrypsin and trypsin) at 20 mM TDBA sodium salt.

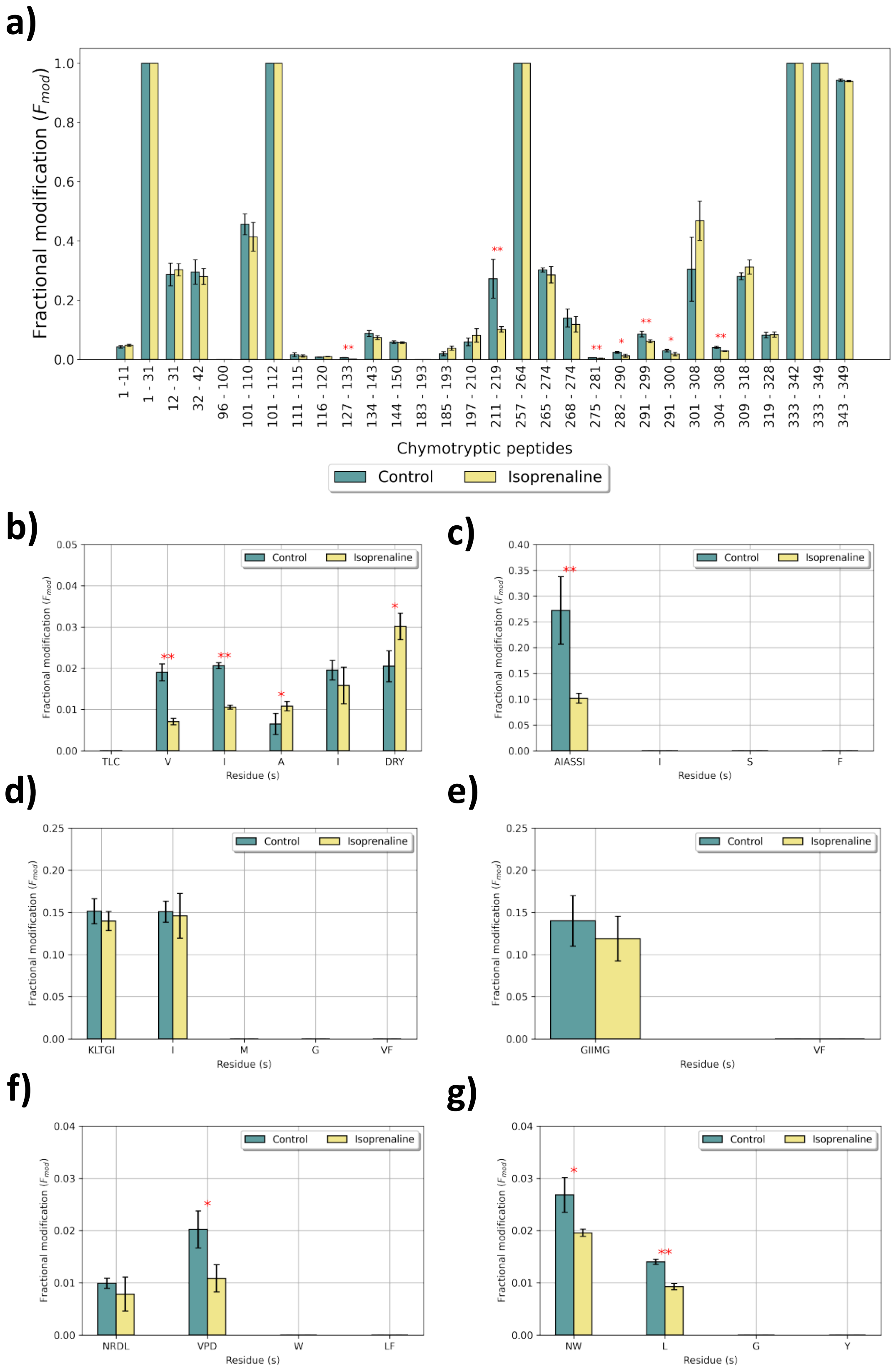

Fig. S12. Carbene footprinting of β1AR with and without isoprenaline using chymotrypsin digestion. (a) Peptide level fractional modification (F_{mod}), sub-peptide F_{mod} from MS/MS of labelled peptides (b) $134^{3.42}$-$143^{3.51}$, (c) $275^{6.45}$-$281^{6.51}$, (d) $265^{6.35}$-$274^{6.44}$, (e) $268^{6.38}$-$274^{6.44}$, (f) $291^{6.61}$-$300^{7.35}$, (g) $304^{7.39}$-$308^{7.43}$. Error bars show ± standard deviation (n = 4). Asterisks denote significant difference between samples (Student t-test; ** = P < 0.01; * = P < 0.05).

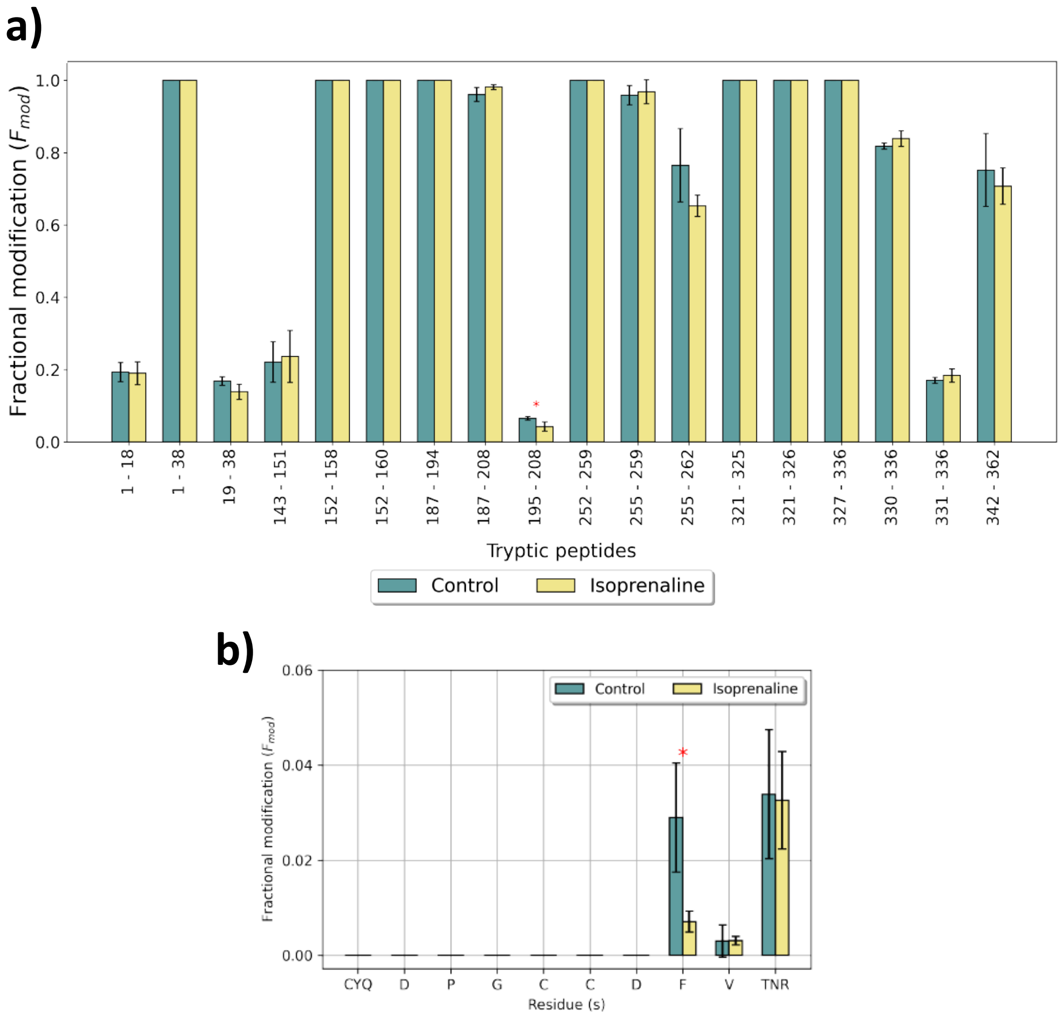

Fig. S13. Carbene footprinting of β1AR with and without isoprenaline using trypsin digestion. (a) Peptide level fractional modification (F_{mod}), (b) sub-peptide F_{mod} from MS/MS of labelled peptide 195-208$^{5.36}$. Error bars show ± standard deviation (n = 4). Asterisks denote significant difference between samples (Student t-test; ** = P < 0.01; * = P < 0.05).

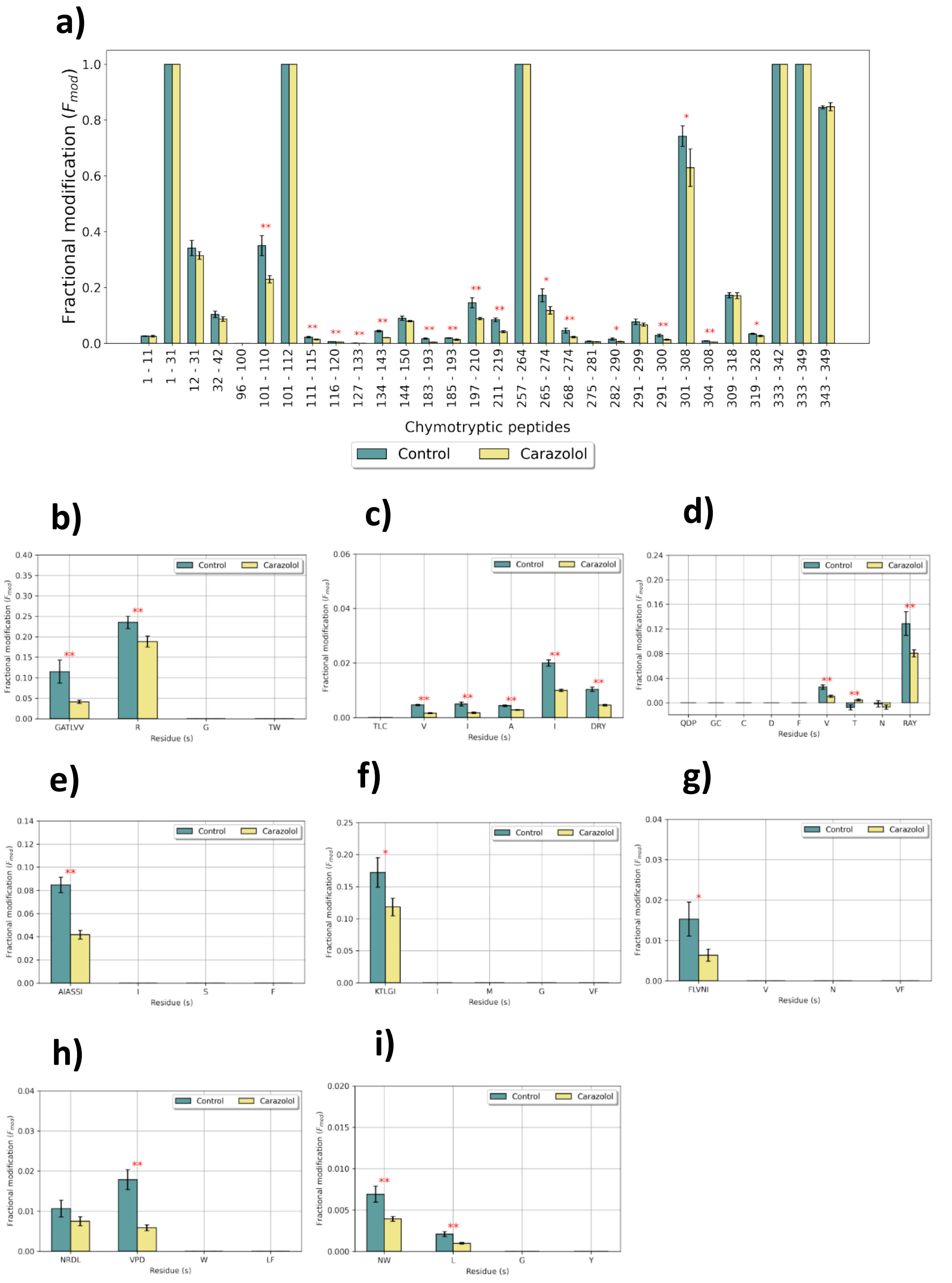

Fig. S14. Carbene footprinting of β1AR with and without carazalol using chymotrypsin digestion. (a) Peptide level fractional modification (F_{mod}), sub-peptide F_{mod} from MS/MS of labelled peptides (b) $101^{2.61}$-$110^{23.50}$, (c) $134^{3.42}$-$143^{3.51}$, (d) 197-$210^{5.38}$, (e) $211^{5.39}$-$219^{5.47}$, (f) $265^{6.35}$-$274^{6.44}$, (g) $282^{6.52}$-$290^{6.60}$, (h) $291^{6.61}$-$299^{7.34}$, (i) $304^{7.39}$-$308^{7.43}$. Error bars show ± standard deviation (n = 4). Asterisks denote significant difference between samples (Student t-test; ** = P < 0.01; * = P < 0.05).

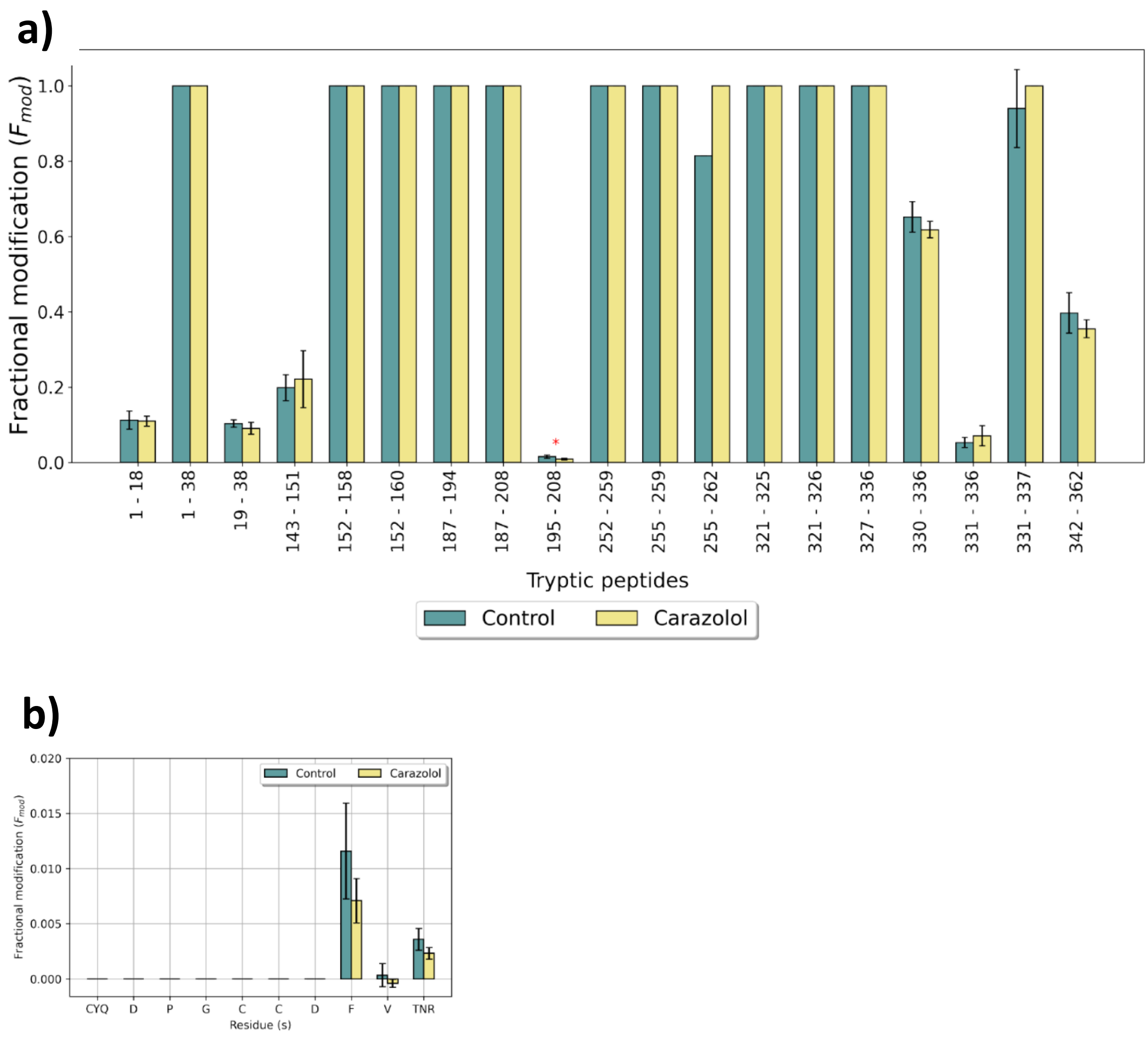

Fig. S15. Carbene footprinting of β1AR with and without carazalol using trypsin digestion. (a) Peptide level fractional modification (F_{mod}), sub-peptide F_{mod} from MS/MS of labelled peptides (b) 195-208[5.36]. Error bars show ± standard deviation (n = 4). Asterisks denote significant difference between samples (Student t-test; ** = P < 0.01; * = P < 0.05).

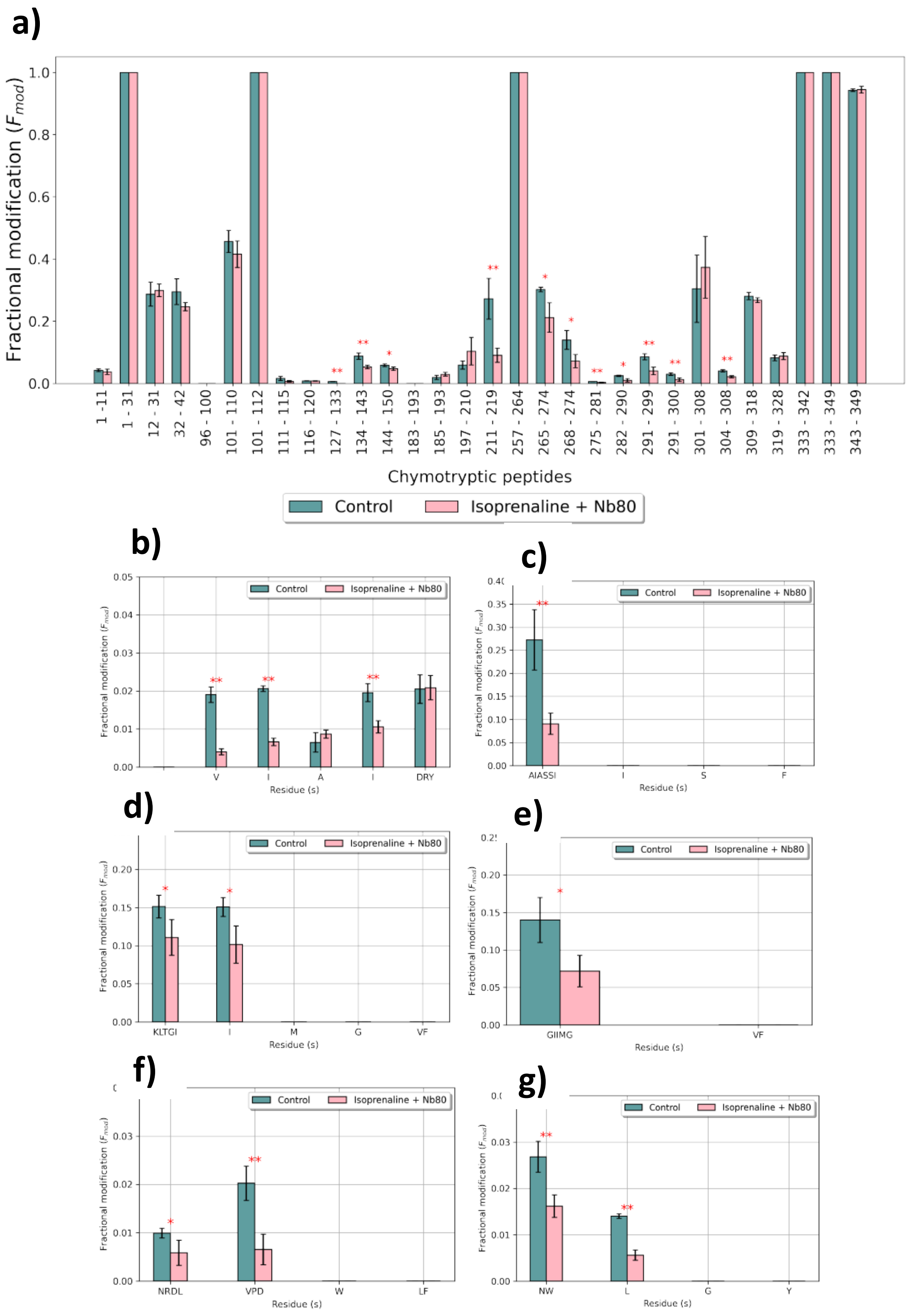

Fig. S16. Carbene footprinting of β1AR with and without isoprenaline + Nb80 using chymotrypsin digestion. (a) Peptide level fractional modification (F_{mod}), sub-peptide F_{mod} from MS/MS of labelled peptides (b) $134^{3.42}$-$143^{3.51}$, (c) $275^{6.45}$-$281^{6.51}$, (d) $265^{6.35}$-$274^{6.44}$, (e) $268^{6.38}$-$274^{6.44}$, (f) $291^{6.61}$-$300^{7.35}$, (g) $304^{7.39}$-$308^{7.43}$. Error bars show ± standard deviation (n = 4). Asterisks denote significant difference between samples (Student t-test; ** = P < 0.01; * = P < 0.05).

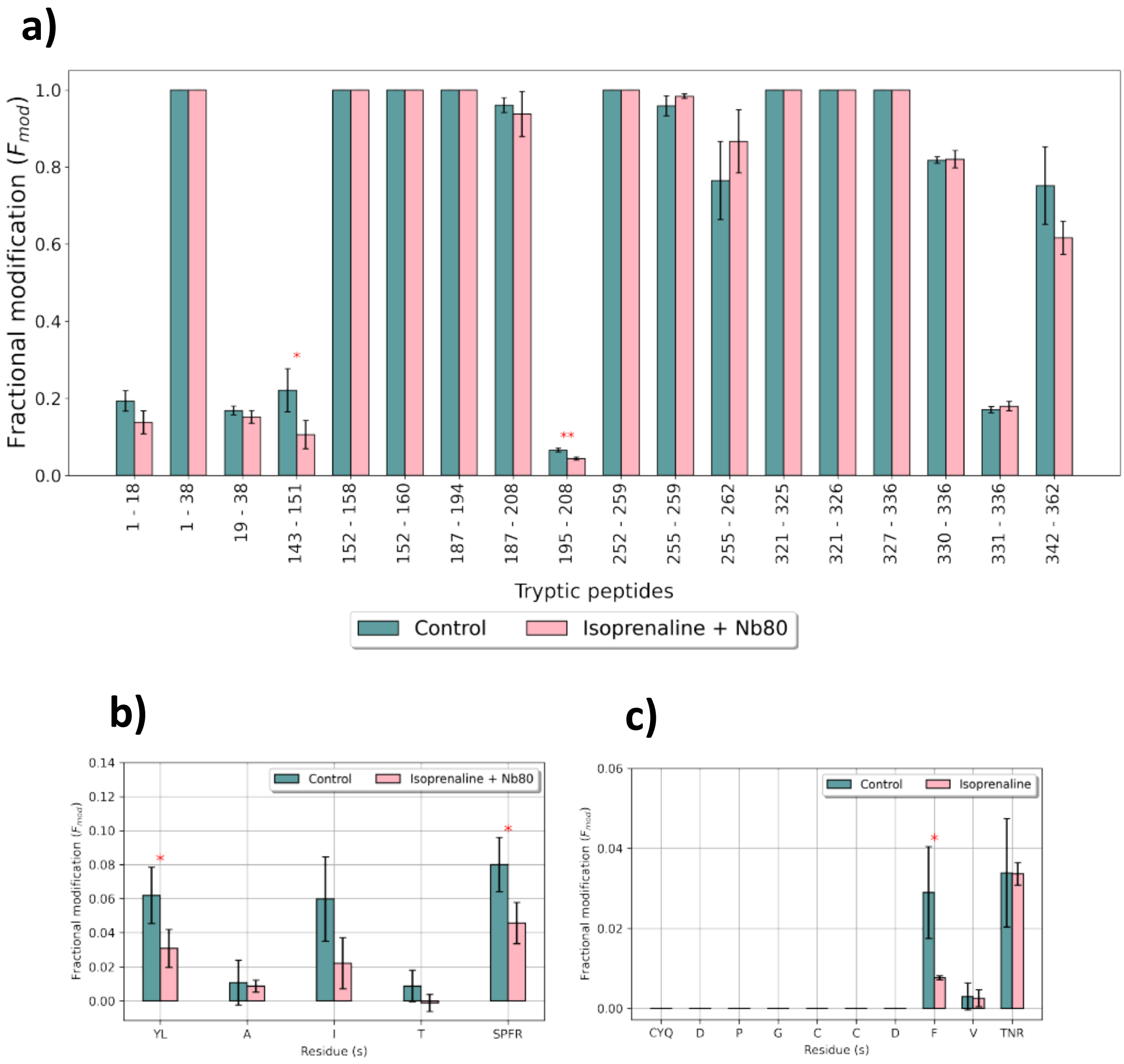

Fig. S17. Carbene footprinting of β1AR with and without isoprenaline + Nb80 using trypsin digestion. (a) Peptide level fractional modification (F_{mod}), (b) sub-peptide F_{mod} from MS/MS of labelled peptide $143^{3.51}$-$151^{34.52}$, (c) sub-peptide F_{mod} from MS/MS of labelled peptide 195-$208^{5.36}$. Error bars show ± standard deviation (n = 4). Asterisks denote significant difference between samples (Student t-test; ** = P < 0.01; * = P < 0.05).

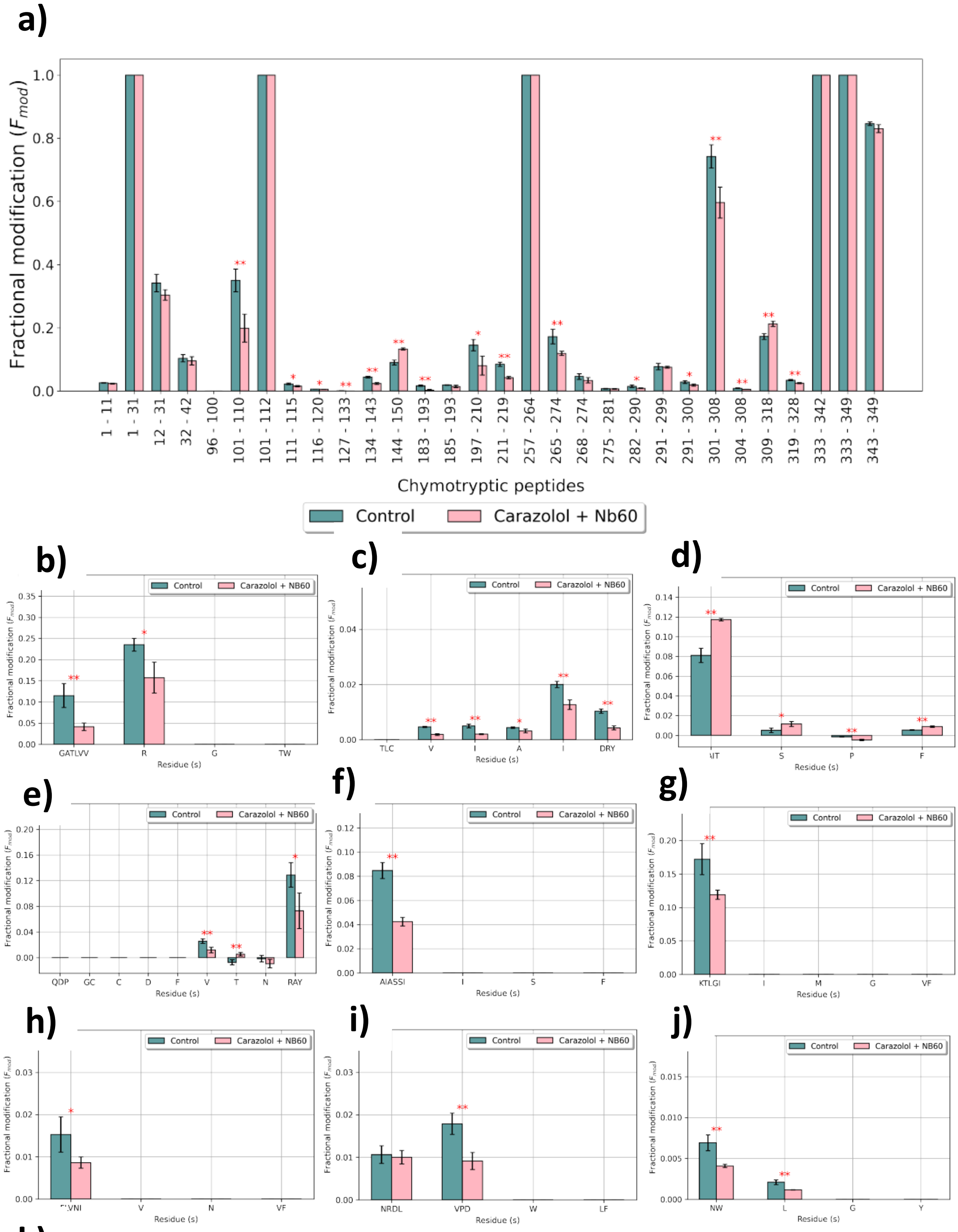

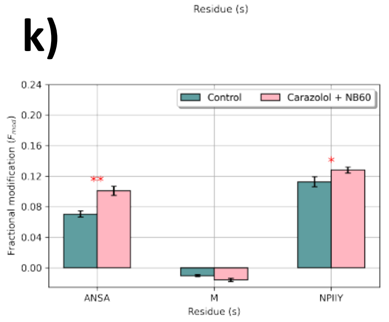

Fig. S18. Carbene footprinting of β1AR with and without carazalol + Nb60 using chymotrypsin digestion. (a) Peptide level fractional modification (F_{mod}), sub-peptide F_{mod} from MS/MS of labelled peptides (b) $101^{2.61}$-$110^{23.50}$, (c) $134^{3.42}$-$143^{3.51}$, (d) $144^{3.52}$-$150^{34.51}$, (e) 197-$210^{5.38}$, (f) $211^{5.39}$-$219^{5.47}$, (g) $265^{6.35}$-$274^{6.44}$, (h) $282^{6.52}$-$290^{6.60}$, (i) $291^{6.61}$-$299^{7.34}$, (j) $304^{7.39}$-$308^{7.43}$, (k) $309^{7.44}$-$318^{7.53}$. Error bars show ± standard deviation (n = 4). Asterisks denote significant difference between samples (Student t-test; ** = $P < 0.01$; * = $P < 0.05$).

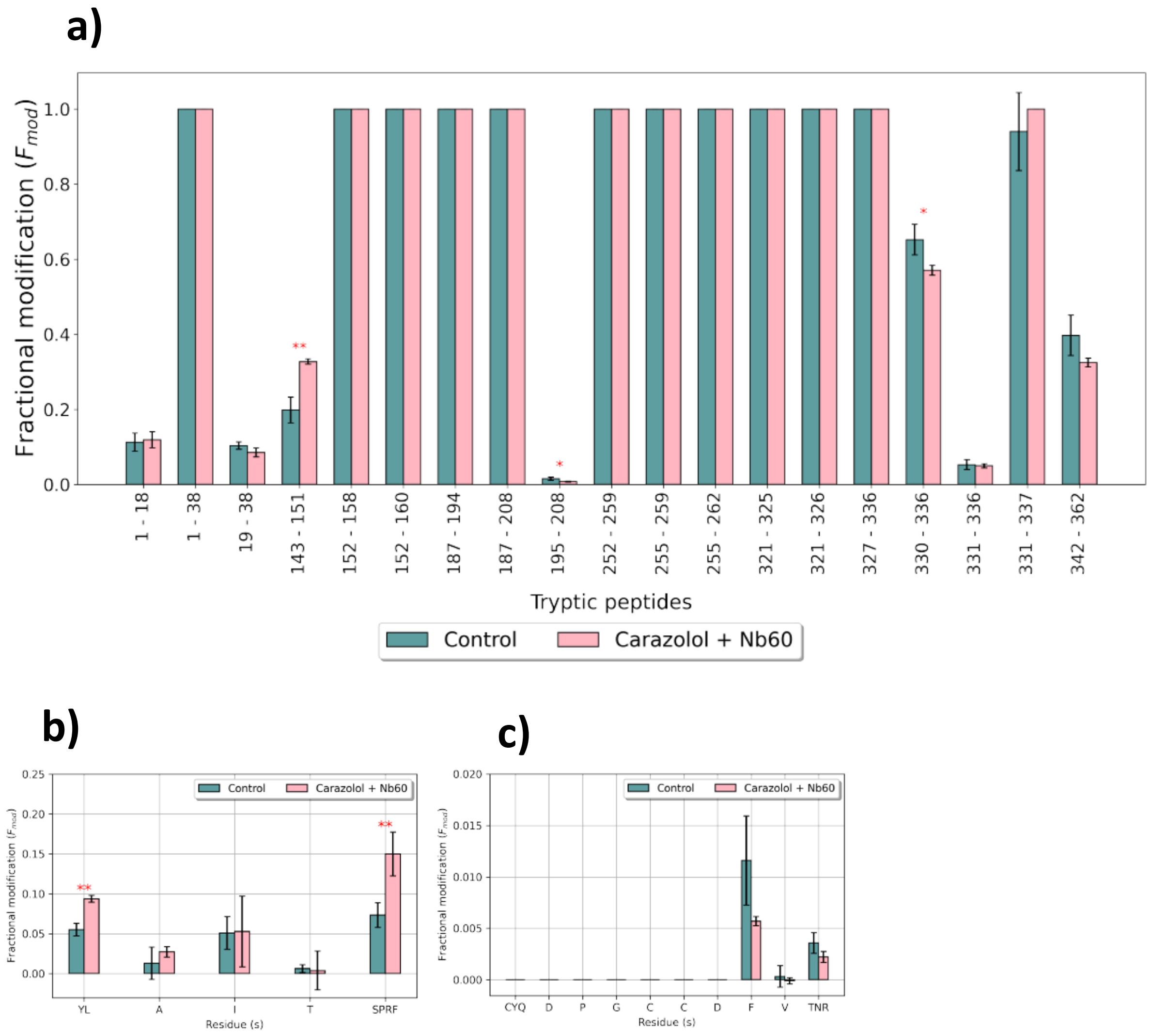

Fig. S19. Carbene footprinting of β1AR with and without carazolol + Nb60 using trypsin digestion. (a) Peptide level fractional modification (F_{mod}), (b) sub-peptide F_{mod} from MS/MS of labelled peptide $143^{3.51}$-$151^{34.52}$, (c) sub-peptide F_{mod} from MS/MS of labelled peptide 195-$208^{5.36}$. Error bars show ± standard deviation (n = 4). Asterisks denote significant difference between samples (Student t-test; ** = P < 0.01; * = P < 0.05).

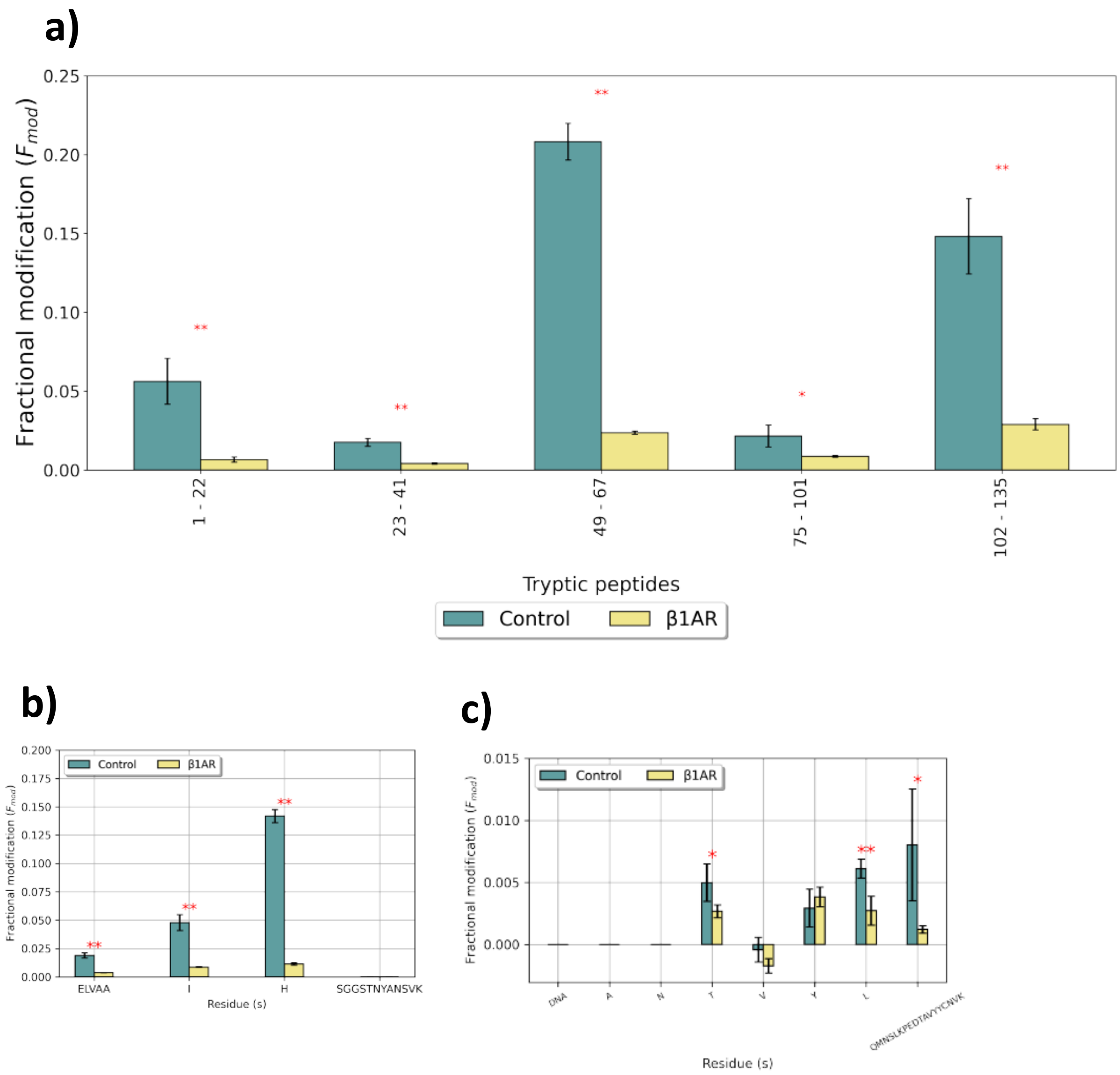

Fig. S20. Carbene footprinting of Nb80 with and without β1AR. (a) Peptide level fractional modification (F_{mod}), (b) sub-peptide F_{mod} from MS/MS of labelled peptide 49-67, (c) sub-peptide F_{mod} from MS/MS of labelled peptide 75-101. Error bars show ± standard deviation (n = 4). Asterisks denote significant difference between samples (Student t-test; ** = P < 0.01; * = P < 0.05).

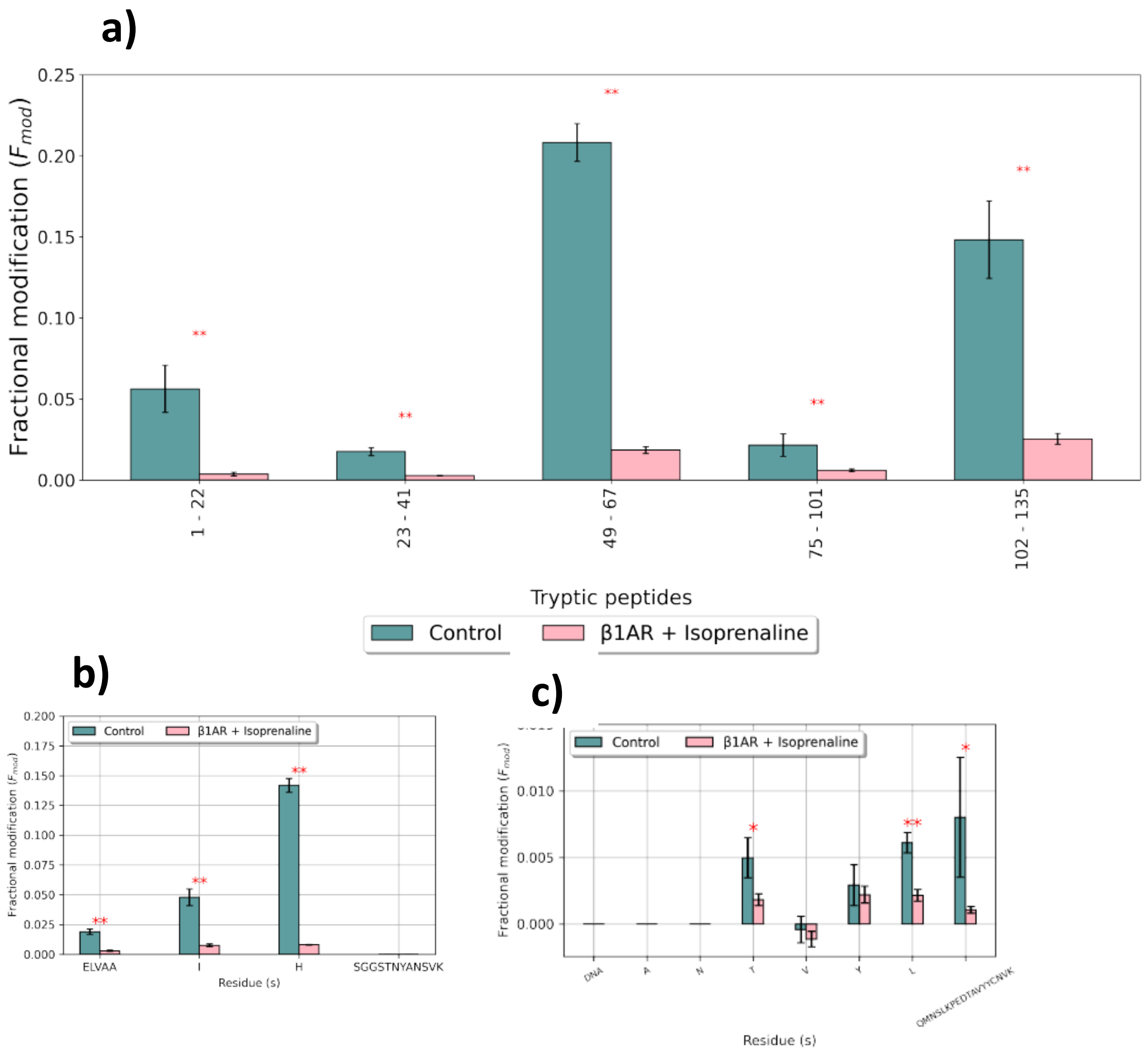

Fig. S21. Carbene footprinting of Nb80 with and without β1AR + isoprenaline. (a) Peptide level fractional modification (F_{mod}), (b) sub-peptide F_{mod} from MS/MS of labelled peptide 49-67, (c) sub-peptide F_{mod} from MS/MS of labelled peptide 75-101. Error bars show ± standard deviation (n = 4). Asterisks denote significant difference between samples (Student t-test; ** = P < 0.01; * = P < 0.05).

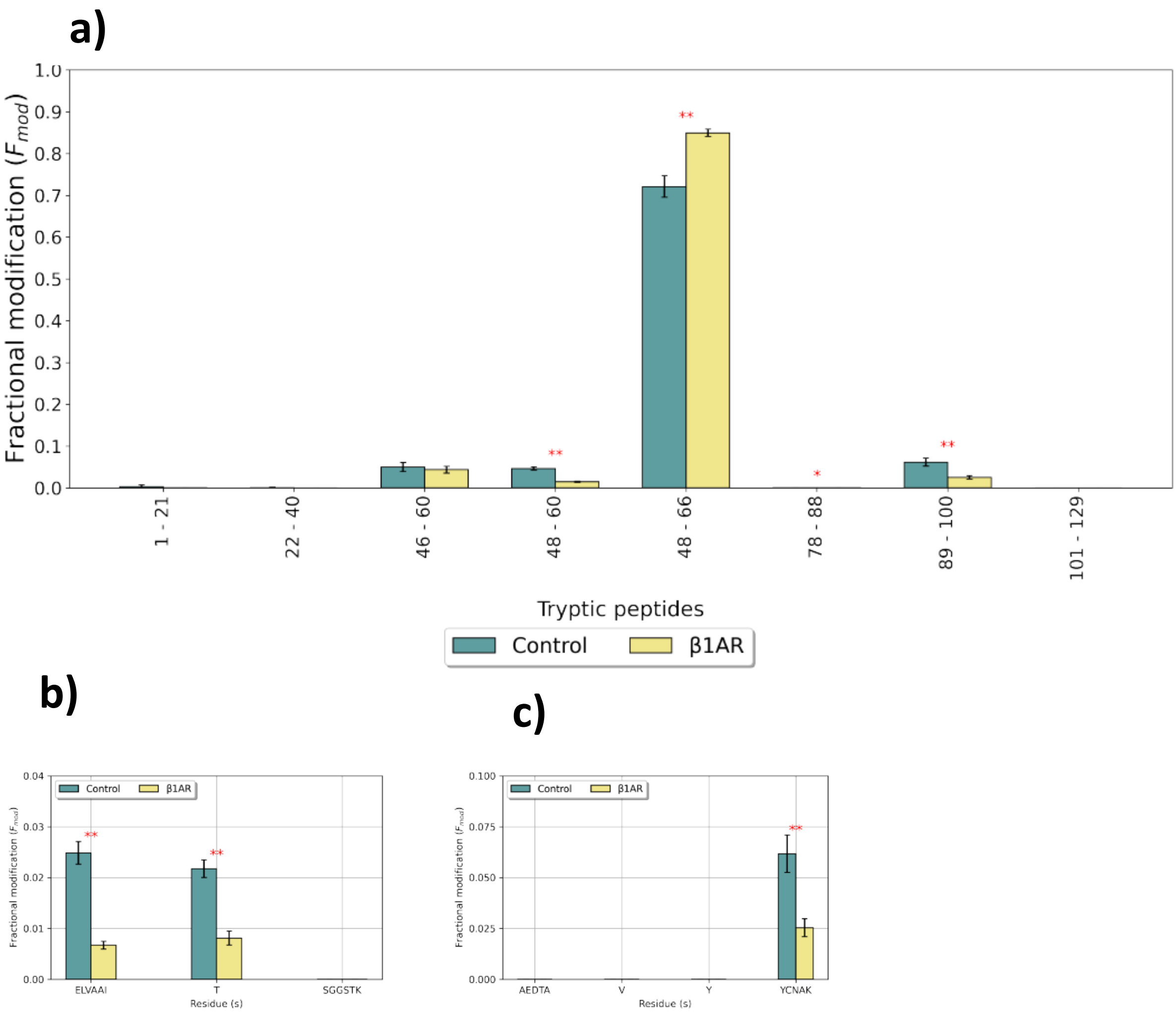

Fig. S22. Carbene footprinting of Nb60 with and without β1AR. (a) Peptide level fractional modification (F_{mod}), (b) sub-peptide F_{mod} from MS/MS of labelled peptide 48-60, (c) sub-peptide F_{mod} from MS/MS of labelled peptide 89-100. Error bars show ± standard deviation (n = 4). Asterisks denote significant difference between samples (Student t-test; ** = P < 0.01; * = P < 0.05).

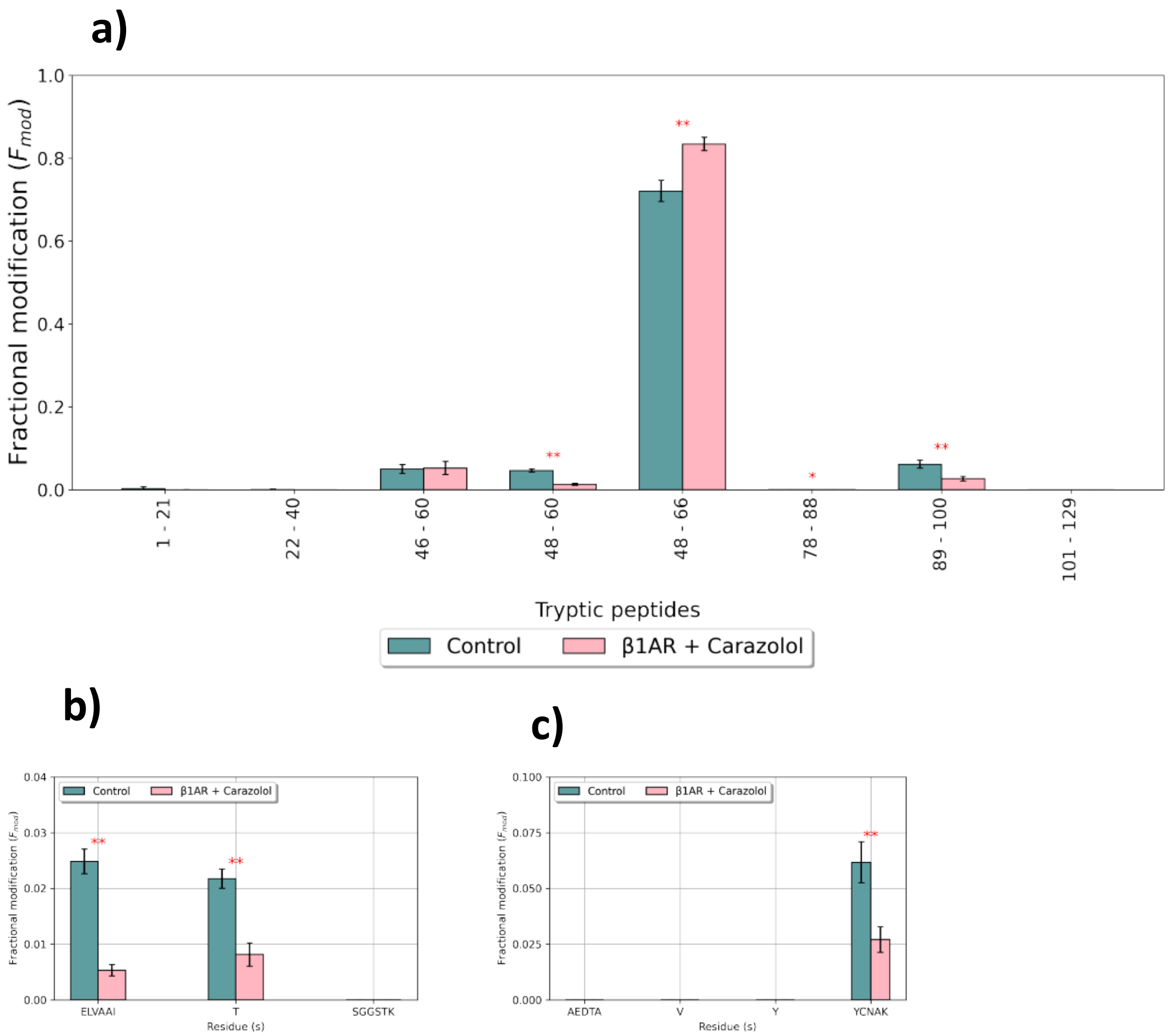

Fig. S23. Carbene footprinting of Nb60 with and without β1AR + carazolol. (a) Peptide level fractional modification (F_{mod}), (b) sub-peptide F_{mod} from MS/MS of labelled peptide 48-60, (c) sub-peptide F_{mod} from MS/MS of labelled peptide 89-100. Error bars show ± standard deviation (n = 4). Asterisks denote significant difference between samples (Student t-test; ** = P < 0.01; * = P < 0.05).

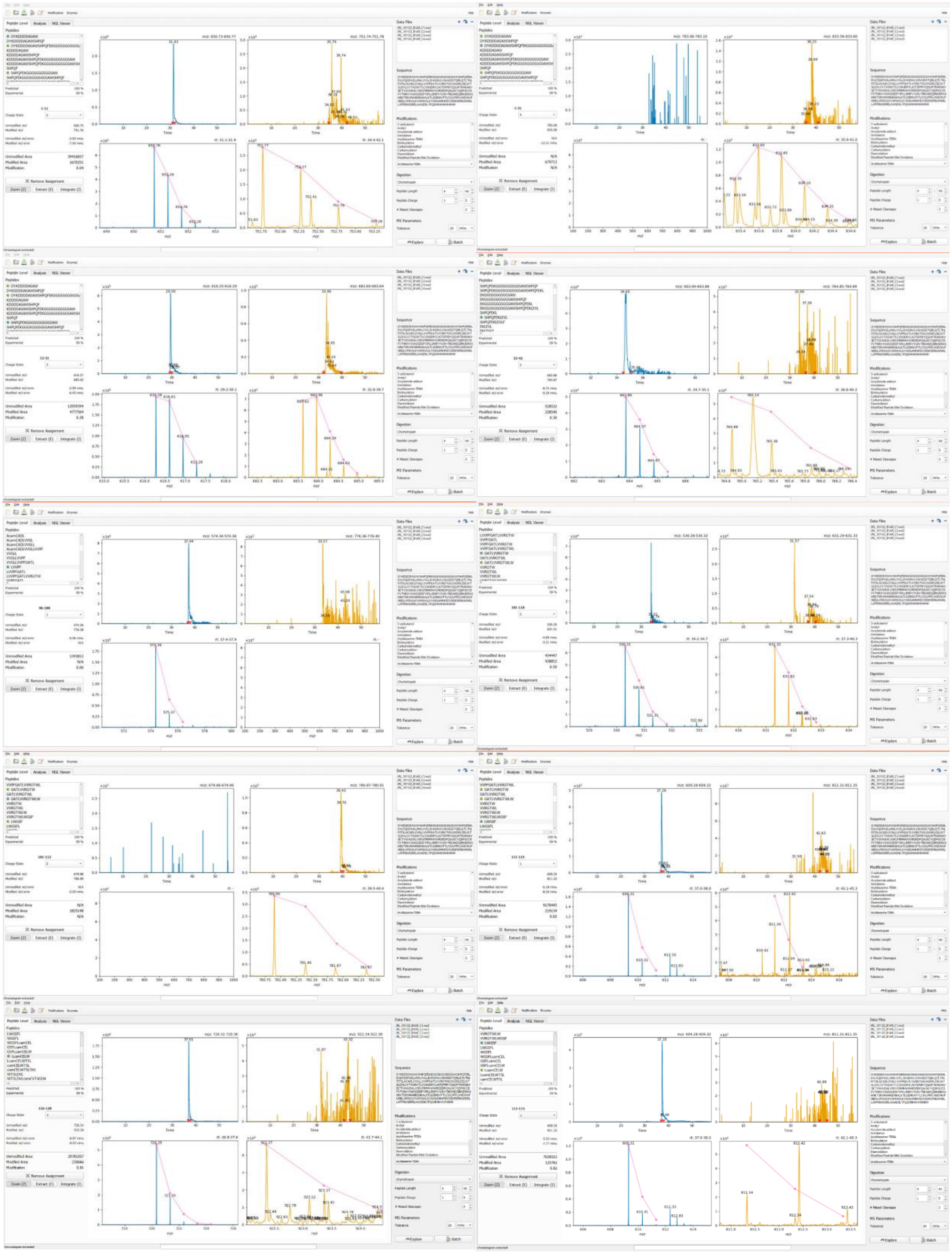

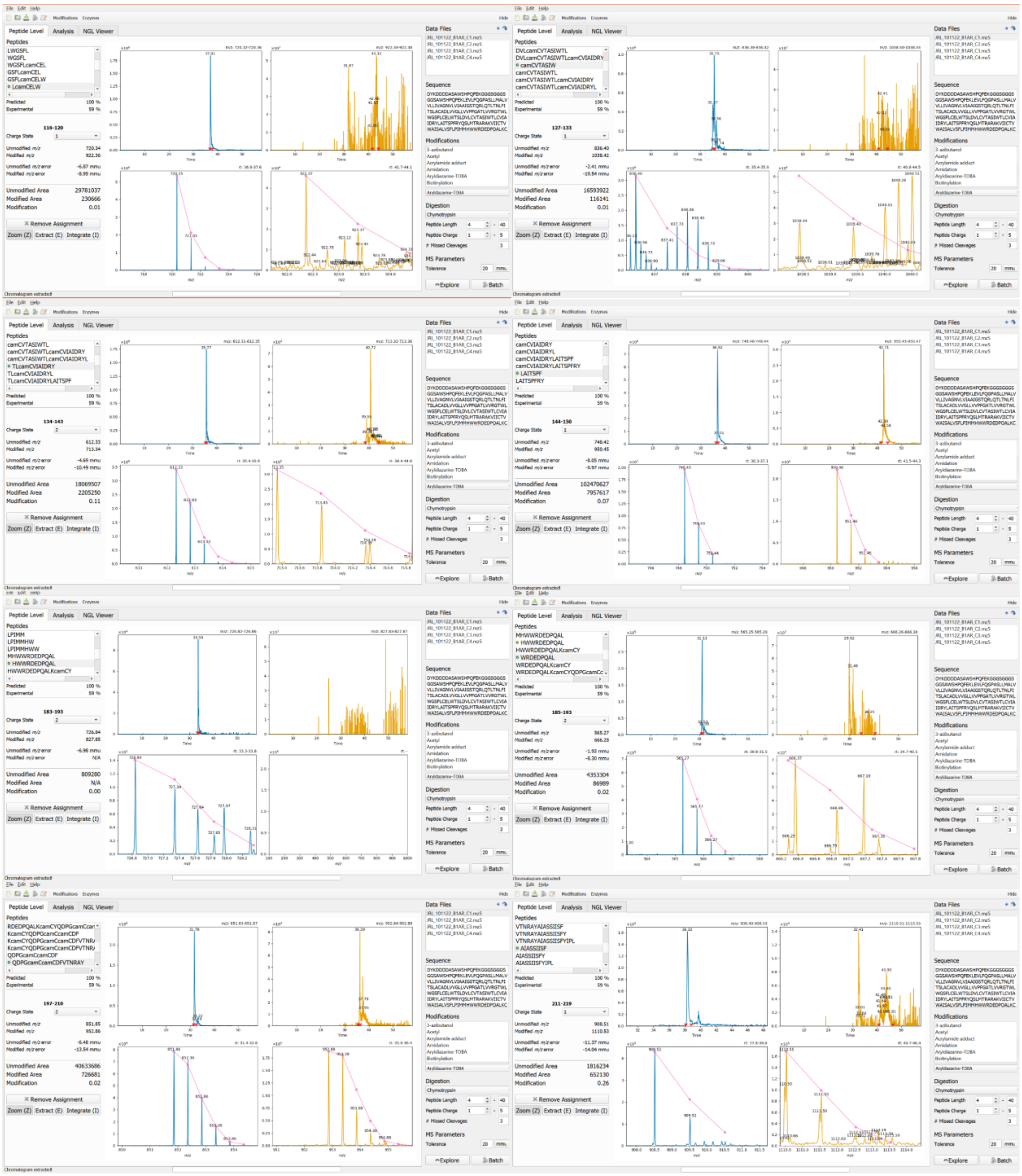

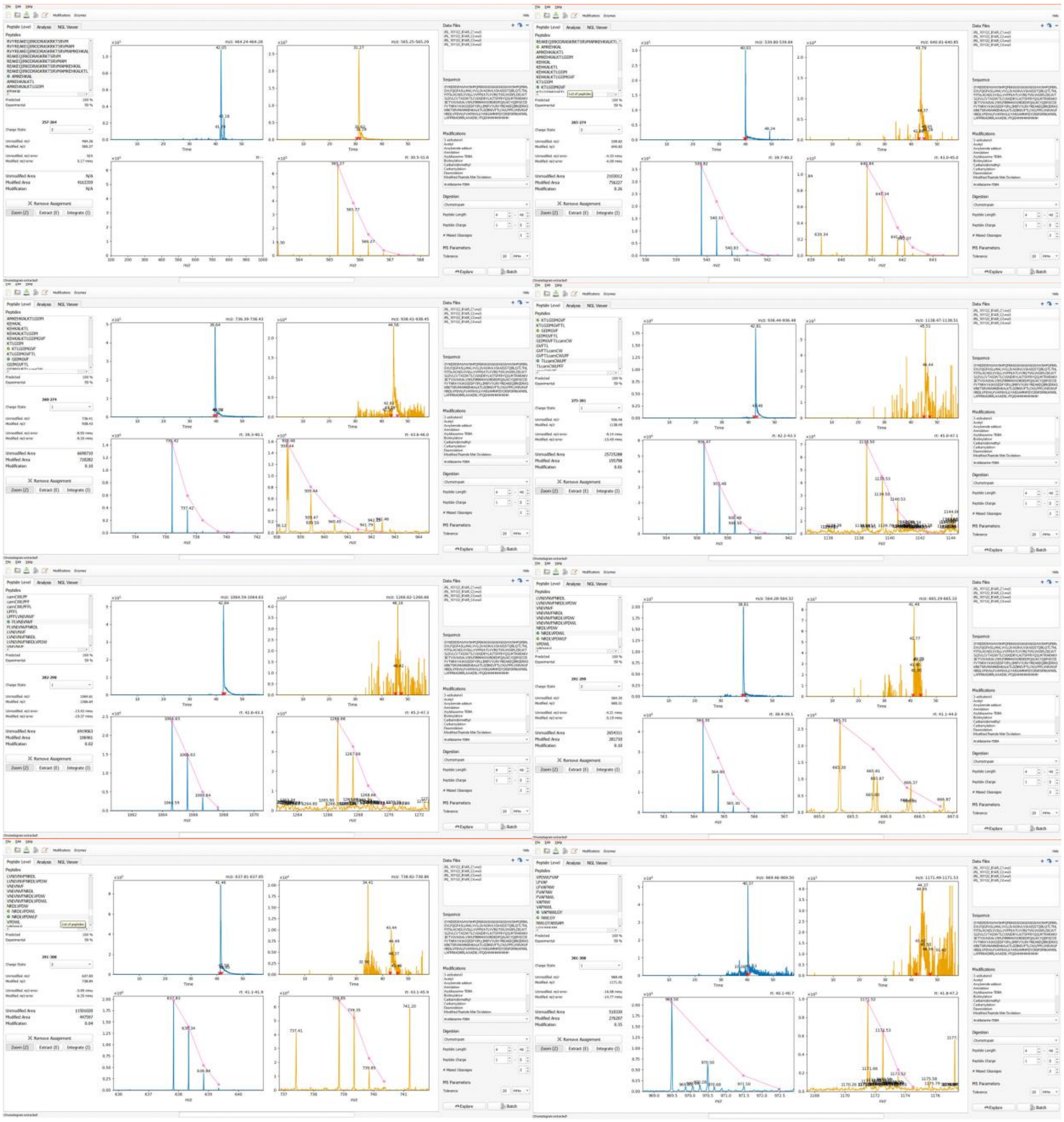

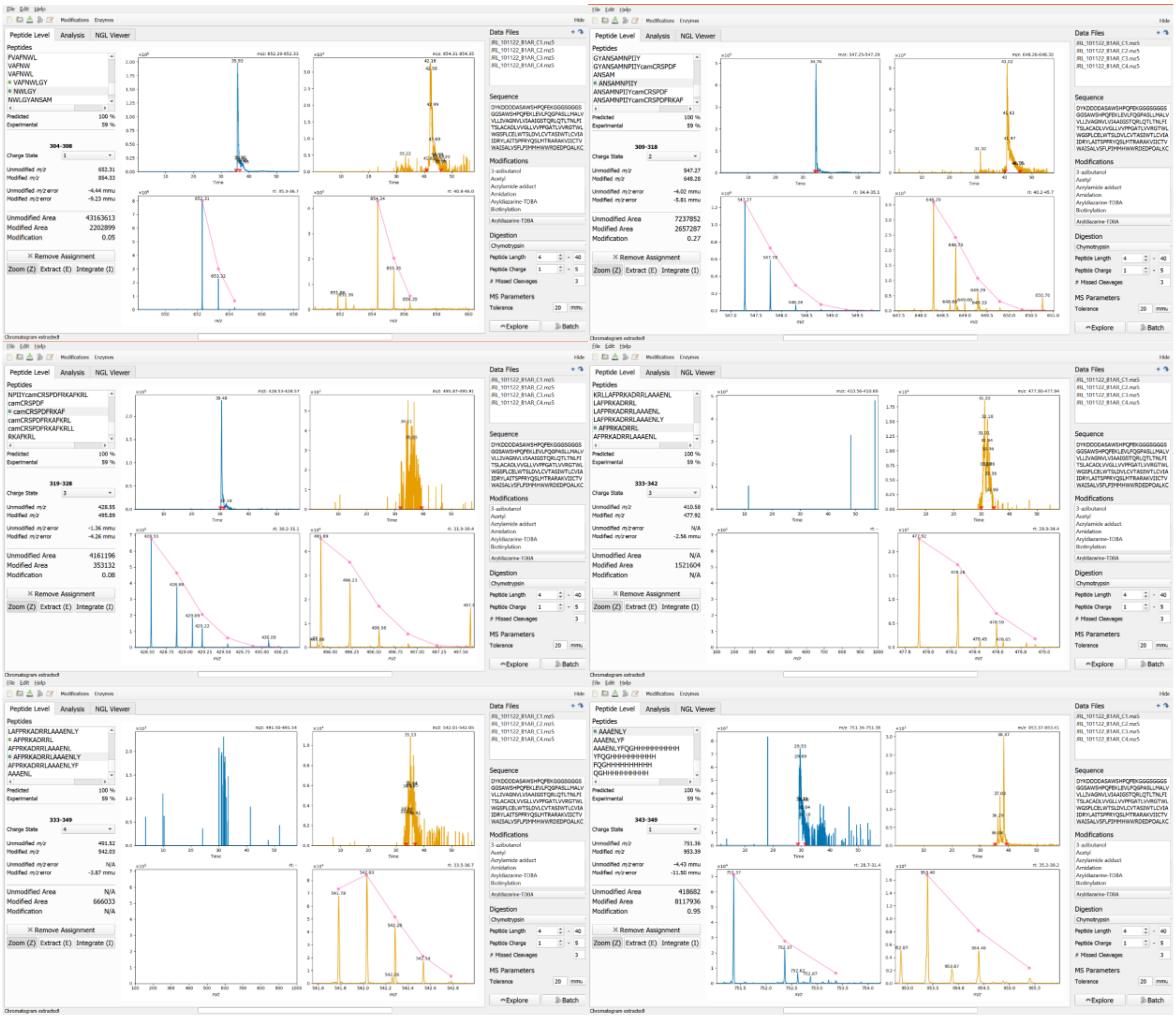

Fig S24: Pepfoot screenshots showing the extracted ion chromatograms for each unlabelled and labelled chymotryptic peptide from one control β1AR sample. The charge state for each peptide, the unlabelled and labelled peptide *m/z* error, the calculated integrated peak area of both unlabelled and labelled peptides and the resulting fractional modification are seen on the left-hand side. Additional information for the search including sequence, fixed and variable modifications, peptide lengths, charge states, and missed cleavages also shown on the right-hand side.

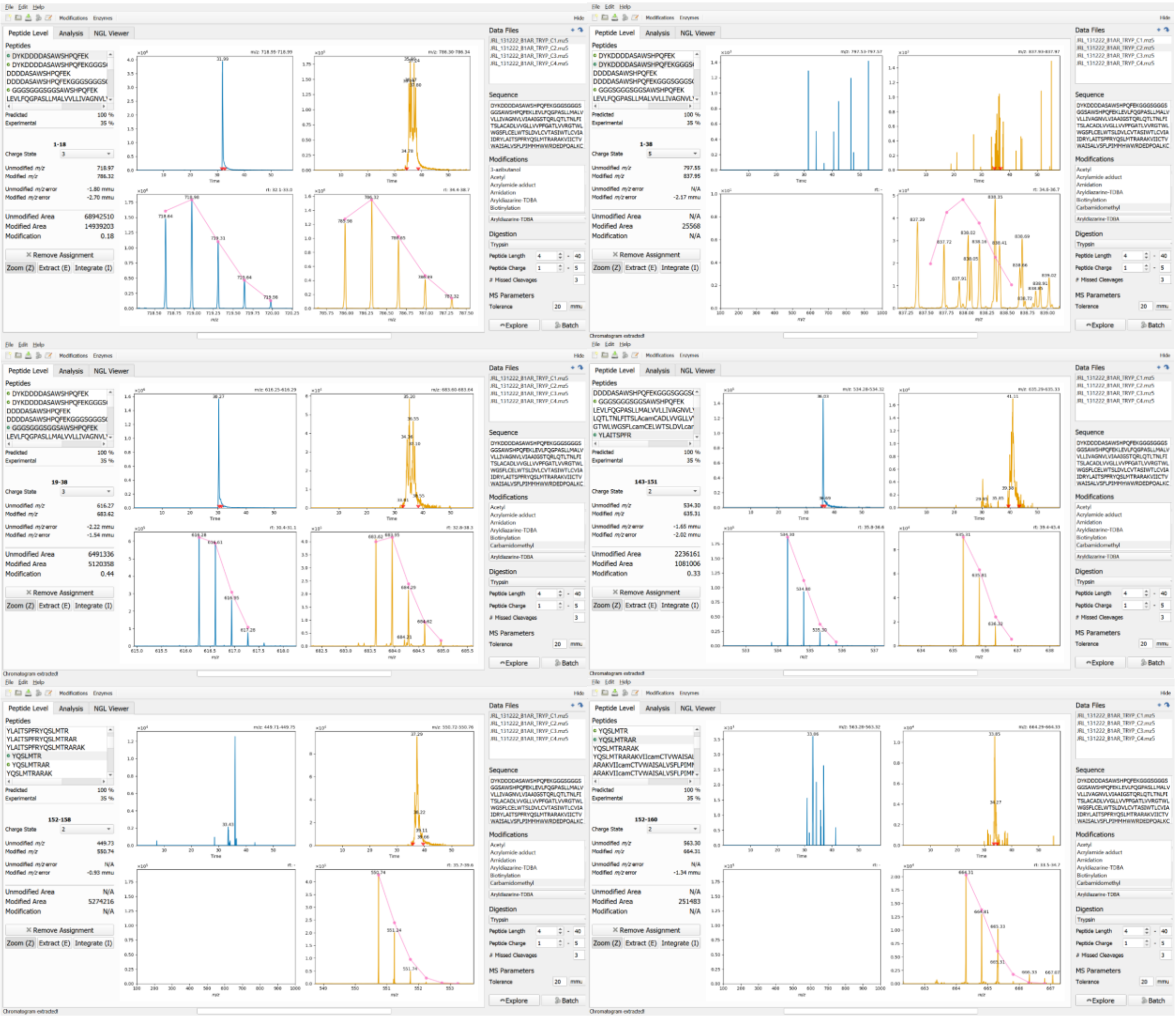

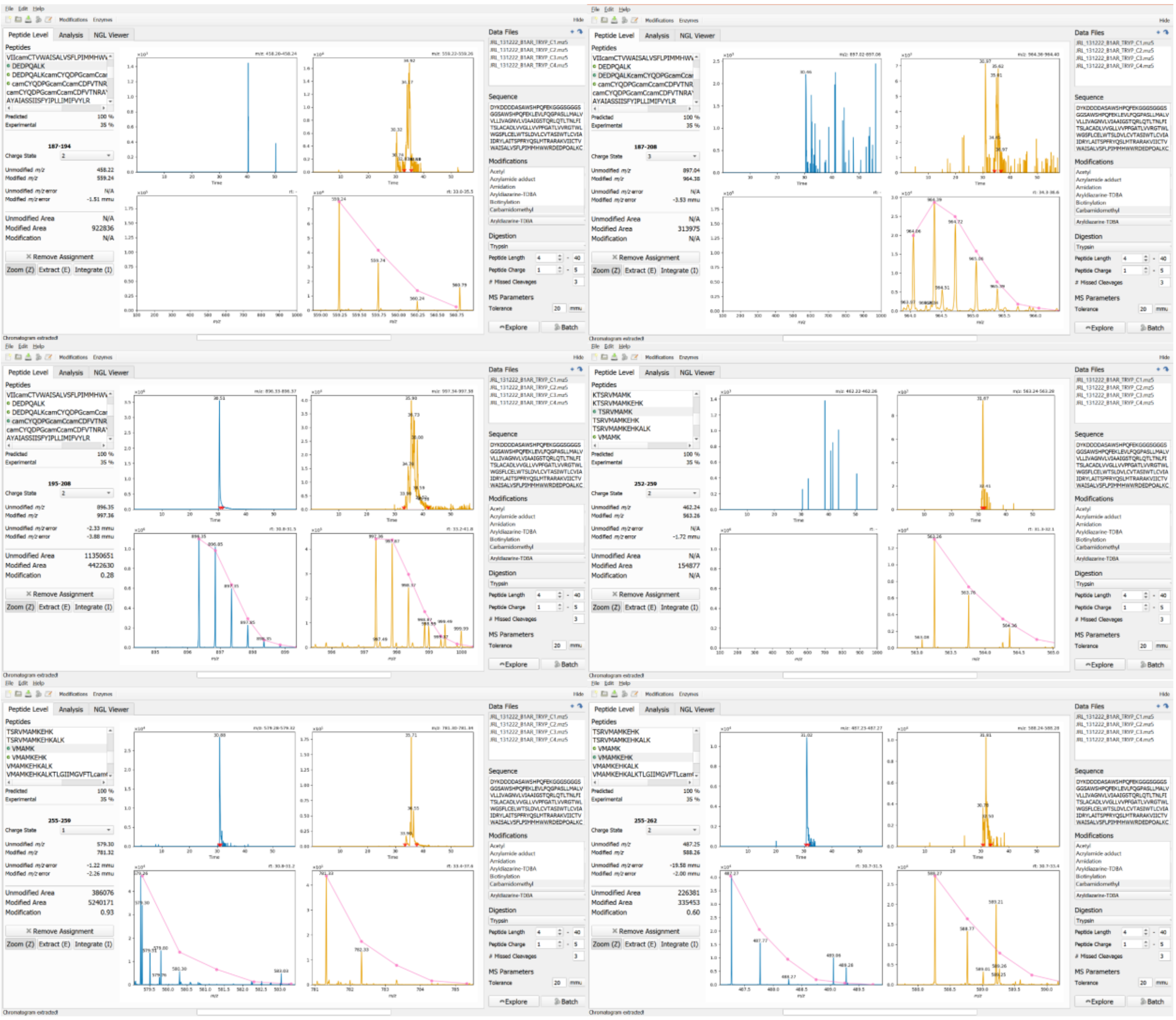

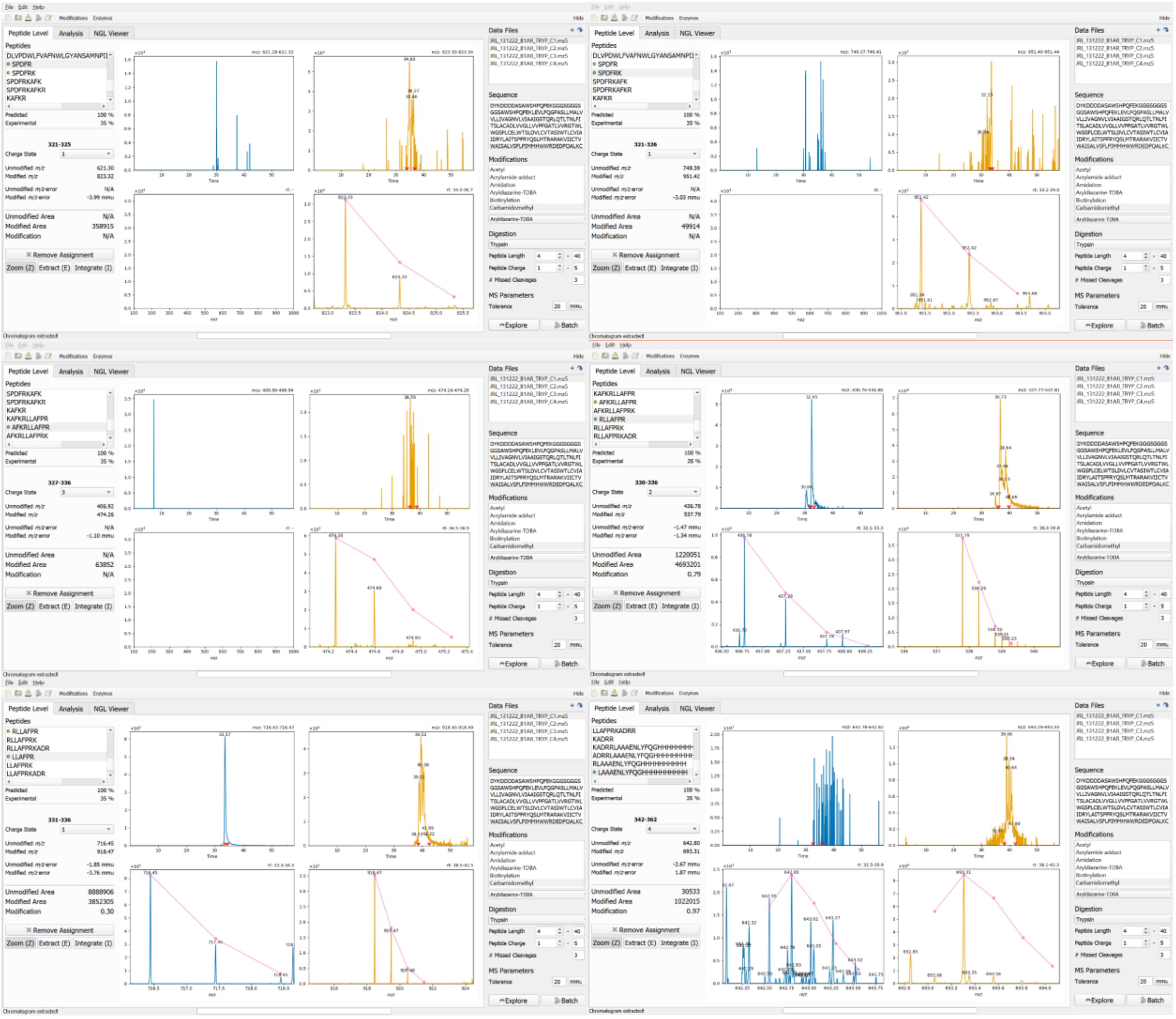

Fig S25: Pepfoot screenshots showing the extracted ion chromatograms for each unlabelled and labelled tryptic peptide from one control β1AR sample. The charge state for each peptide, the unlabelled and labelled peptide *m/z* error, the calculated integrated peak area of both unlabelled and labelled peptides and the resulting fractional modification are seen on the left-hand side. Additional information for the search including sequence, fixed and variable modifications, peptide lengths, charge states, and missed cleavages also shown on the right-hand side.

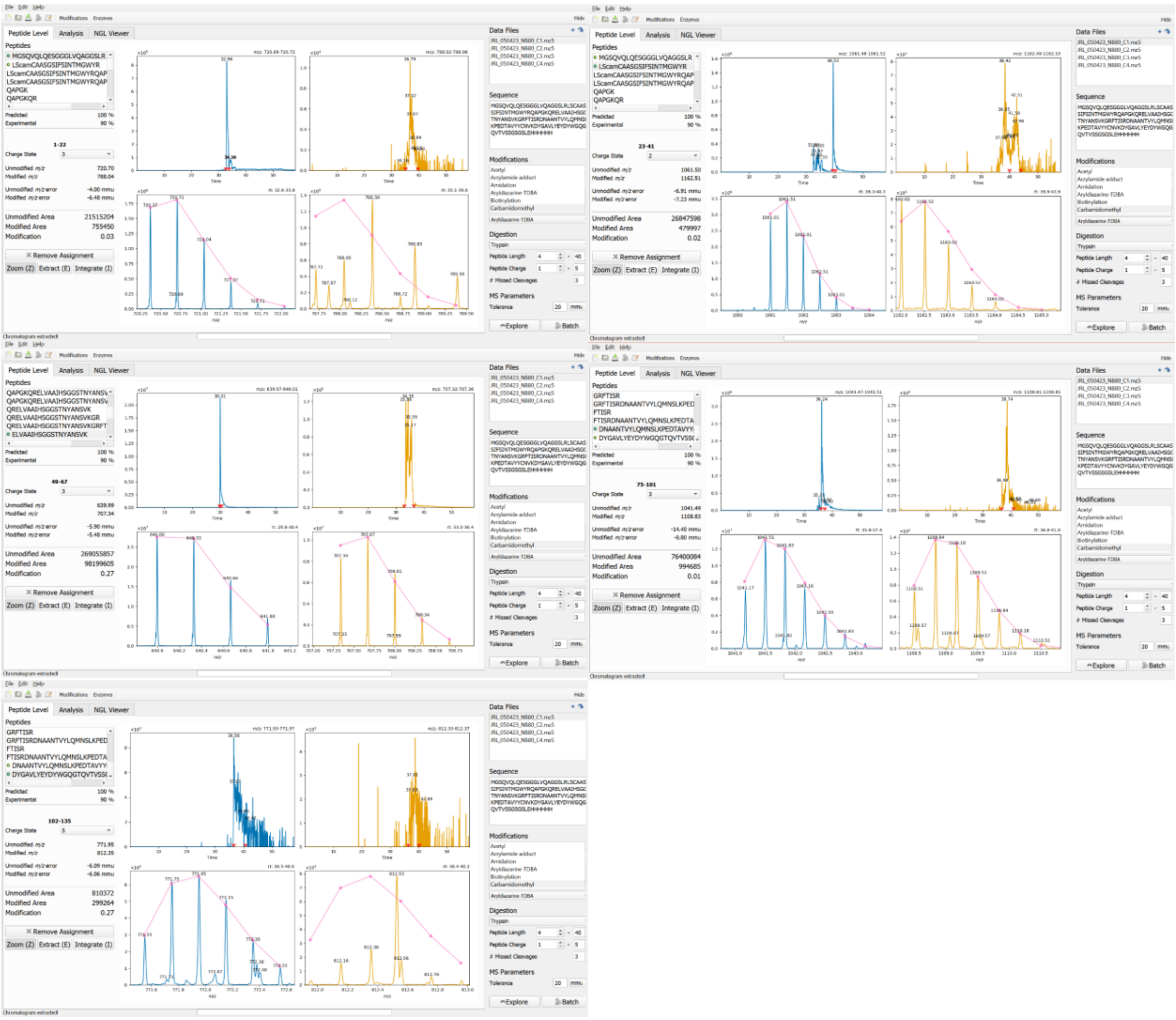

Fig S26: Pepfoot screenshots showing the extracted ion chromatograms for each unlabelled and labelled tryptic peptide from one control Nb80 sample. The charge state for each peptide, the unlabelled and labelled peptide *m/z* error, the calculated integrated peak area of both unlabelled and labelled peptides and the resulting fractional modification are seen on the left-hand side. Additional information for the search including sequence, fixed and variable modifications, peptide lengths, charge states, and missed cleavages also shown on the right-hand side.

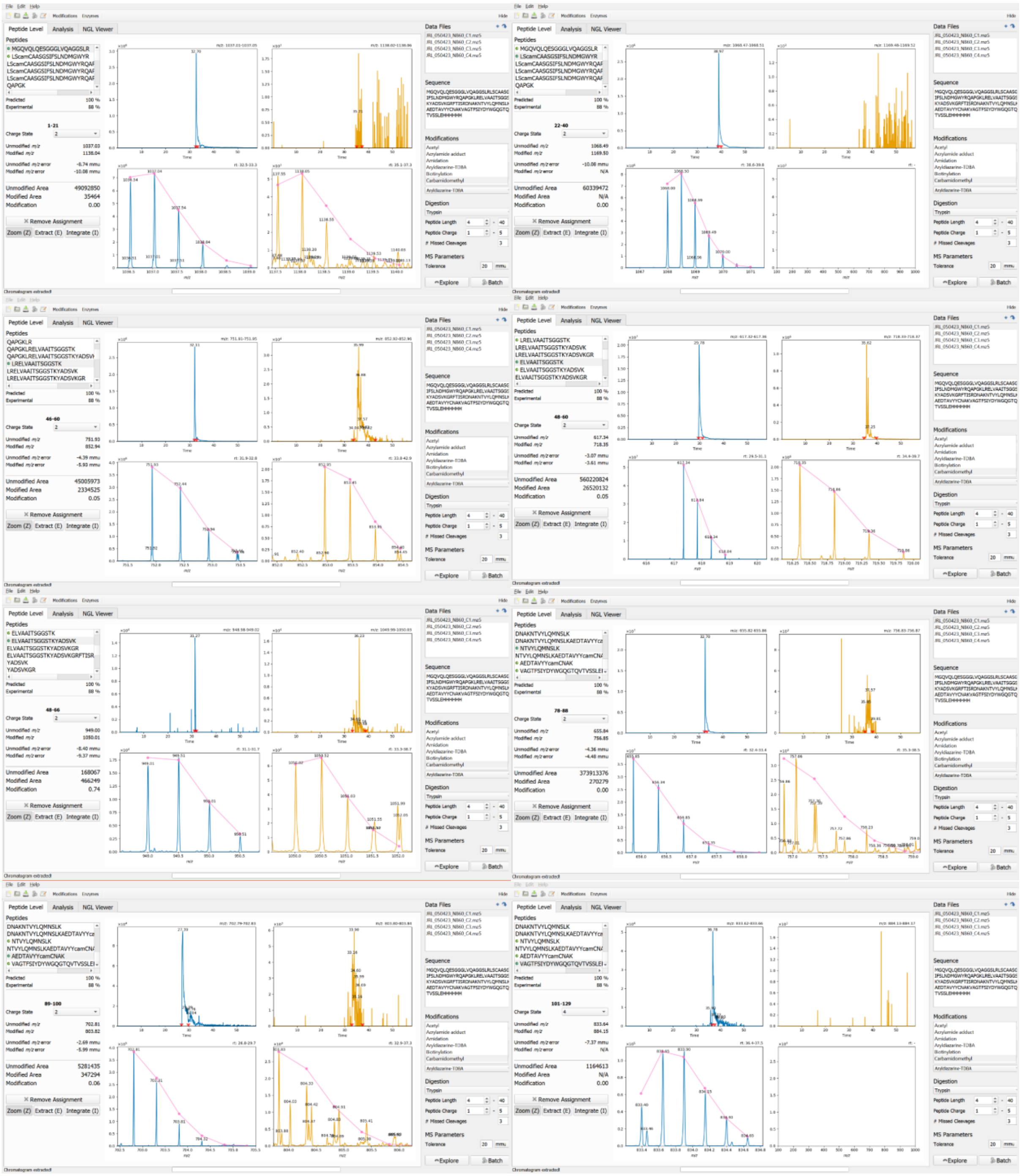

Fig S27: Pepfoot screenshots showing the extracted ion chromatograms for each unlabelled and labelled tryptic peptide from one control Nb60 sample. The charge state for each peptide, the unlabelled and labelled peptide *m/z* error, the calculated integrated peak area of both unlabelled and labelled peptides and the resulting fractional modification are seen on the left-hand side. Additional information for the search including sequence, fixed and variable modifications, peptide lengths, charge states, and missed cleavages also shown on the right-hand side.

Table S1: Fractional modification values of chymotryptic β1AR peptides in the absence and presence of isoprenaline and isoprenaline + Nb80.

		Control				+ Isoprenaline				+ Isoprenaline +Nb80				Control		+ Isoprenaline		+ Isoprenaline +Nb80	
Peptide No.	Peptide	1	2	3	4	1	2	3	4	1	2	3	4	Mean	St.D	Mean	St.D	Mean	St.D
1- 11	DYKDDDDASAW	0.035	0.045	0.046	0.043	0.044	0.050	0.045	0.053	0.052	0.033	0.035	0.030	0.042	0.004	0.048	0.004	0.037	0.009
1 - 31	DYKDDDDASAWSHPQFEKGGGSGGGSGGSAW	1.000	1.000	1.000	1.000	1.000	1.000	1.000	1.000	1.000	1.000	1.000	1.000	1.000	0.000	1.000	0.000	1.000	0.000
12 - 31	SHPQFEKGGGSGGGSGGSAW	0.233	0.272	0.335	0.308	0.282	0.337	0.291	0.302	0.296	0.310	0.323	0.269	0.287	0.038	0.303	0.021	0.299	0.020
32 - 42	SHPQFEKLEVL	0.250	0.261	0.351	0.318	0.311	0.301	0.263	0.245	0.265	0.240	0.253	0.230	0.295	0.041	0.280	0.027	0.247	0.013
96 - 100	LVVPF	0.000	0.000	0.000	0.000	0.000	0.000	0.000	0.000	0.000	0.000	0.000	0.000	0.000	0.000	0.000	0.000	0.000	0.000
101 - 110	GATLVVRGTW	0.408	0.445	0.466	0.506	0.430	0.484	0.354	0.389	0.436	0.429	0.344	0.454	0.456	0.035	0.414	0.049	0.416	0.043
101 - 112	GATLVVRGTWLW	1.000	1.000	1.000	1.000	1.000	1.000	1.000	1.000	1.000	1.000	1.000	1.000	1.000	0.000	1.000	0.000	1.000	0.000
111 - 115	LWGSF	0.012	0.009	0.026	0.017	0.016	0.015	0.007	0.009	0.003	0.008	0.008	0.010	0.016	0.006	0.012	0.004	0.007	0.003
116 - 120	LCELW	0.008	0.007	0.009	0.009	0.009	0.010	0.010	0.012	0.007	0.009	0.007	0.009	0.008	0.001	0.010	0.001	0.008	0.001
127 - 133	CVTASIW	0.007	0.005	0.007	0.006	0.001	0.001	0.002	0.001	0.000	0.000	0.000	0.000	0.006	0.001	0.001	0.000	0.000	0.000
134 - 143	TLCVIAIDRY	0.084	0.073	0.099	0.095	0.080	0.072	0.080	0.064	0.061	0.046	0.053	0.053	0.088	0.010	0.074	0.007	0.053	0.005
144 - 150	LAITSPF	0.058	0.052	0.063	0.062	0.057	0.059	0.052	0.060	0.040	0.049	0.047	0.054	0.059	0.004	0.057	0.003	0.047	0.005
183 - 193	HWWRDEDPQAL	0.000	0.000	0.000	0.000	0.000	0.000	0.000	0.000	0.000	0.000	0.000	0.000	0.000	0.000	0.000	0.000	0.000	0.000
185 - 193	WRDEDPQAL	0.020	0.008	0.022	0.027	0.049	0.037	0.034	0.032	0.032	0.027	0.022	0.038	0.019	0.007	0.038	0.007	0.030	0.006
197 - 210	QDPGCCDFVTNRAY	0.054	0.041	0.073	0.070	0.112	0.095	0.063	0.057	0.094	0.093	0.053	0.175	0.059	0.013	0.082	0.023	0.104	0.044
211 - 219	AIASSIISF	0.274	0.376	0.242	0.198	0.092	0.115	0.094	0.106	0.064	0.091	0.127	0.081	0.272	0.065	0.102	0.009	0.091	0.023
257 - 264	AMKEHKAL	1.000	1.000	1.000	1.000	1.000	1.000	1.000	1.000	1.000	1.000	1.000	1.000	1.000	0.000	1.000	0.000	1.000	0.000
265 - 274	KTLGIIMGVF	0.311	0.304	0.291	0.303	0.260	0.331	0.266	0.286	0.142	0.243	0.198	0.266	0.302	0.007	0.286	0.028	0.212	0.047
268 - 274	GIIMGVF	0.135	0.190	0.113	0.122	0.104	0.128	0.087	0.157	0.043	0.089	0.060	0.095	0.140	0.030	0.119	0.026	0.072	0.021
275 - 281	TLCWLPF	0.006	0.007	0.006	0.006	0.004	0.004	0.003	0.006	0.002	0.004	0.002	0.005	0.006	0.000	0.004	0.001	0.003	0.001
282 - 290	FLVNIVNVF	0.024	0.022	0.026	0.027	0.008	0.018	0.007	0.019	0.005	0.016	0.004	0.015	0.025	0.002	0.013	0.005	0.010	0.006
291 - 299	NRDLVPDWL	0.076	0.076	0.095	0.096	0.064	0.060	0.055	0.069	0.026	0.044	0.033	0.058	0.086	0.010	0.062	0.005	0.040	0.012
291 - 300	NRDLVPDWLF	0.034	0.024	0.028	0.034	0.015	0.018	0.014	0.028	0.006	0.019	0.007	0.017	0.030	0.004	0.019	0.006	0.012	0.006
301 - 308	VAFNWLGY	0.269	0.153	0.349	0.448	0.501	0.552	0.448	0.373	0.273	0.469	0.275	0.476	0.305	0.108	0.468	0.066	0.373	0.099
304 - 308	NWLGY	0.043	0.035	0.045	0.041	0.030	0.027	0.029	0.030	0.017	0.024	0.021	0.025	0.041	0.004	0.029	0.001	0.022	0.003
309 - 318	ANSAMNPIIY	0.263	0.277	0.293	0.289	0.325	0.319	0.333	0.272	0.278	0.258	0.271	0.264	0.281	0.012	0.312	0.024	0.268	0.008
319 - 328	CRSPDFRKAF	0.069	0.092	0.091	0.078	0.092	0.070	0.084	0.089	0.104	0.086	0.089	0.073	0.082	0.009	0.084	0.008	0.088	0.011
333 - 342	AFPRKADRRL	1.000	1.000	1.000	1.000	1.000	1.000	1.000	1.000	1.000	1.000	1.000	1.000	1.000	0.000	1.000	0.000	1.000	0.000
333 - 349	AFPRKADRRLAAAENLY	1.000	1.000	1.000	1.000	1.000	1.000	1.000	1.000	1.000	1.000	1.000	1.000	1.000	0.000	1.000	0.000	1.000	0.000
343 - 349	AAAENLY	0.949	0.942	0.938	0.941	0.941	0.940	0.941	0.936	0.962	0.939	0.933	0.945	0.943	0.004	0.939	0.002	0.945	0.011

Table S2: Fractional modification values of tryptic β1AR peptides in the absence and presence of isoprenaline and isoprenaline + Nb80.

		Control				+ Isoprenaline				+ Isoprenaline +Nb80				Control		+ Isoprenaline		+ Isoprenaline +Nb80	
Peptide No.	Peptide	1	2	3	4	1	2	3	4	1	2	3	4	Mean	St.D	Mean	St.D	Mean	St.D
1 - 18	DYKDDDDASAWSHPQFEK	0.222	0.188	0.211	0.153	0.207	0.228	0.143	0.183	0.146	0.166	0.152	0.088	0.193	0.026	0.190	0.032	0.138	0.030
1 - 38	DYKDDDDASAWSHPQFEKGGGSGGGSGGSAWSHPQFEK	1.000	1.000	1.000	1.000	1.000	1.000	1.000	1.000	1.000	1.000	1.000	1.000	1.000	0.000	1.000	0.000	1.000	0.000
19 - 38	GGGSGGGSGGSAWSHPQFEK	0.183	0.153	0.177	0.163	0.135	0.147	0.167	0.108	0.173	0.162	0.138	0.133	0.169	0.012	0.139	0.021	0.152	0.016
143 - 151	YLAITSPFR	0.296	0.195	0.248	0.146	0.220	0.308	0.127	0.292	0.125	0.121	0.134	0.043	0.221	0.056	0.237	0.072	0.106	0.037
152 - 158	YQSLMTR	1.000	1.000	1.000	1.000	1.000	1.000	1.000	1.000	1.000	1.000	1.000	1.000	1.000	0.000	1.000	0.000	1.000	0.000
152 - 160	YQSLMTRAR	1.000	1.000	1.000	1.000	1.000	1.000	1.000	1.000	1.000	1.000	1.000	1.000	1.000	0.000	1.000	0.000	1.000	0.000
187 - 194	DEDPQALK	1.000	1.000	1.000	1.000	1.000	1.000	1.000	1.000	1.000	1.000	1.000	1.000	1.000	0.000	1.000	0.000	1.000	0.000
187 - 208	DEDPQALKCYQDPGCCDFVTNR	0.958	0.958	0.991	0.936	0.988	0.985	0.982	0.971	0.977	0.974	0.962	0.837	0.961	0.019	0.981	0.006	0.938	0.058
195 - 208	CYQDPGCCDFVTNR	0.073	0.066	0.065	0.059	0.053	0.053	0.043	0.022	0.049	0.044	0.045	0.038	0.066	0.005	0.043	0.013	0.044	0.004
252 - 259	TSRVMAMK	1.000	1.000	1.000	1.000	1.000	1.000	1.000	1.000	1.000	1.000	1.000	1.000	1.000	0.000	1.000	0.000	1.000	0.000
255 - 259	VMAMK	0.917	0.991	0.963	0.963	0.980	0.988	0.911	0.994	0.985	0.993	0.978	0.980	0.959	0.026	0.968	0.033	0.984	0.006
255 - 262	VMAMKEHK	0.602	0.875	0.813	0.770	0.661	0.654	0.690	0.608	0.928	0.963	0.813	0.762	0.765	0.101	0.653	0.029	0.867	0.082
321 - 325	SPDFR	1.000	1.000	1.000	1.000	1.000	1.000	1.000	1.000	1.000	1.000	1.000	1.000	1.000	0.000	1.000	0.000	1.000	0.000
321 - 326	SPDFRK	1.000	1.000	1.000	1.000	1.000	1.000	1.000	1.000	1.000	1.000	1.000	1.000	1.000	0.000	1.000	0.000	1.000	0.000
327 - 336	AFKRLLAFPR	1.000	1.000	1.000	1.000	1.000	1.000	1.000	1.000	1.000	1.000	1.000	1.000	1.000	0.000	1.000	0.000	1.000	0.000
330 - 336	RLLAFPR	0.823	0.816	0.827	0.805	0.843	0.863	0.804	0.846	0.824	0.837	0.838	0.782	0.818	0.008	0.839	0.022	0.820	0.023
331 - 336	LLAFPR	0.184	0.166	0.170	0.163	0.176	0.215	0.179	0.167	0.191	0.182	0.187	0.159	0.171	0.008	0.184	0.018	0.180	0.012
342 - 362	LAAAENLYFQGHHHHHHHHHH	0.919	0.742	0.674	0.672	0.679	0.643	0.773	0.736	0.673	0.628	0.612	0.553	0.752	0.101	0.707	0.050	0.616	0.043

Table S3: Fractional modification values of tryptic Nb80 peptides in the absence and presence of β1AR and β1AR + isoprenaline.

		Control				+ B1AR				+ B1AR + Isoprenaline				Control		+ B1AR		+ B1AR + Isoprenaline	
Peptide No.	Peptide	1	2	3	4	1	2	3	4	1	2	3	4	Mean	St.D	Mean	St.D	Mean	St.D
1 - 22	MGSQVQLQESGGGLVQAGGSLR	0.040	0.044	0.075	0.065	0.007	0.009	0.006	0.005	0.005	0.005	0.003	0.003	0.056	0.015	0.007	0.002	0.004	0.001
23 - 41	LSCAASGSIFSINTMGWYR	0.015	0.017	0.022	0.017	0.004	0.005	0.005	0.004	0.003	0.003	0.002	0.003	0.018	0.002	0.004	0.000	0.003	0.000
49 - 67	ELVAAIHSGGSTNYANSVK	0.226	0.201	0.212	0.196	0.025	0.025	0.022	0.023	0.021	0.020	0.018	0.015	0.208	0.012	0.024	0.001	0.019	0.002
75 - 101	DNAANTVYLQMNSLKPEDTAVYYCNVK	0.017	0.016	0.033	0.021	0.009	0.009	0.008	0.008	0.006	0.007	0.005	0.007	0.022	0.007	0.009	0.001	0.006	0.001
102 - 135	DYGAVLYEYDYWGQGTQVTVSSGSGSLEHHHHHH	0.179	0.117	0.162	0.135	0.031	0.031	0.023	0.031	0.027	0.020	0.028	0.027	0.148	0.024	0.029	0.004	0.025	0.003

Table S4: Fractional modification values of chymotryptic β1AR peptides in the absence and presence of carazolol and carazolol + Nb60.

		Control				+ Carazolol				+ Carazolol + Nb60				Control		+ Carazolol		+ Carazolol + Nb60	
Peptide No.	Peptide	1	2	3	4	1	2	3	4	1	2	3	4	Mean	St.D	Mean	St.D	Mean	St.D
1- 11	DYKDDDDASAW	0.025	0.026	0.027	0.026	0.022	0.031	0.027	0.024	0.025	0.023	0.025	0.021	0.026	0.001	0.026	0.003	0.024	0.002
1 - 31	DYKDDDDASAWSHPQFEKGGGSGGGSGGSAW	1.000	1.000	1.000	1.000	1.000	1.000	1.000	1.000	1.000	1.000	1.000	1.000	1.000	0.000	1.000	0.000	1.000	0.000
12 - 31	SHPQFEKGGGSGGGSGGSAW	0.304	0.373	0.361	0.329	0.304	0.314	0.338	0.304	0.306	0.301	0.327	0.281	0.342	0.027	0.315	0.014	0.304	0.017
32 - 42	SHPQFEKLEVL	0.123	0.094	0.102	0.097	0.074	0.093	0.089	0.094	0.096	0.083	0.116	0.086	0.104	0.011	0.087	0.008	0.096	0.013
96 - 100	LVVPF	0.000	0.000	0.000	0.000	0.000	0.000	0.000	0.000	0.000	0.000	0.000	0.000	0.000	0.000	0.000	0.000	0.000	0.000
101 - 110	GATLVVRGTW	0.364	0.315	0.403	0.319	0.218	0.215	0.243	0.242	0.225	0.141	0.255	0.174	0.350	0.036	0.229	0.013	0.199	0.044
101 - 112	GATLVVRGTWLW	1.000	1.000	1.000	1.000	1.000	1.000	1.000	1.000	1.000	1.000	1.000	1.000	1.000	0.000	1.000	0.000	1.000	0.000
111 - 115	LWGSF	0.019	0.021	0.025	0.026	0.014	0.014	0.017	0.013	0.015	0.014	0.019	0.014	0.023	0.003	0.014	0.001	0.016	0.002
116 - 120	LCELW	0.005	0.006	0.006	0.007	0.005	0.005	0.005	0.005	0.005	0.006	0.006	0.005	0.006	0.001	0.005	0.000	0.006	0.000
127 - 133	CVTASIW	0.001	0.001	0.001	0.001	0.000	0.000	0.000	0.000	0.000	0.000	0.000	0.000	0.001	0.000	0.000	0.000	0.000	0.000
134 - 143	TLCVIAIDRY	0.049	0.044	0.042	0.042	0.021	0.021	0.021	0.020	0.020	0.022	0.028	0.026	0.044	0.003	0.021	0.000	0.024	0.003
144 - 150	LAITSPF	0.079	0.090	0.100	0.093	0.081	0.077	0.084	0.077	0.136	0.135	0.127	0.134	0.090	0.008	0.080	0.003	0.133	0.003
183 - 193	HWWRDEDPQAL	0.017	0.016	0.015	0.020	0.005	0.004	0.006	0.004	0.005	0.000	0.000	0.005	0.017	0.002	0.005	0.001	0.003	0.003
185 - 193	WRDEDPQAL	0.021	0.019	0.018	0.020	0.011	0.014	0.018	0.012	0.018	0.009	0.018	0.013	0.019	0.001	0.014	0.003	0.014	0.004
197 - 210	QDPGCCDFVTNRAY	0.169	0.141	0.152	0.119	0.086	0.084	0.095	0.091	0.101	0.044	0.118	0.061	0.145	0.018	0.089	0.004	0.081	0.030
211 - 219	AIASSIISF	0.075	0.083	0.088	0.093	0.045	0.036	0.045	0.041	0.040	0.047	0.038	0.045	0.085	0.007	0.042	0.004	0.042	0.004
257 - 264	AMKEHKAL	1.000	1.000	1.000	1.000	1.000	1.000	1.000	1.000	1.000	1.000	1.000	1.000	1.000	0.000	1.000	0.000	1.000	0.000
265 - 274	KTLGIIMGVF	0.141	0.161	0.202	0.185	0.117	0.099	0.137	0.120	0.125	0.113	0.127	0.111	0.172	0.023	0.118	0.014	0.119	0.007
268 - 274	GIIMGVF	0.029	0.049	0.047	0.056	0.025	0.019	0.026	0.021	0.032	0.044	0.021	0.039	0.045	0.010	0.023	0.003	0.034	0.009
275 - 281	TLCWLPF	0.006	0.008	0.009	0.008	0.006	0.005	0.006	0.006	0.008	0.008	0.005	0.008	0.008	0.001	0.006	0.001	0.007	0.001
282 - 290	FLVNIVNVF	0.010	0.013	0.020	0.019	0.009	0.005	0.006	0.006	0.010	0.009	0.007	0.009	0.015	0.004	0.006	0.001	0.009	0.001
291 - 299	NRDLVPDWL	0.063	0.077	0.080	0.091	0.060	0.063	0.072	0.073	0.072	0.077	0.080	0.073	0.077	0.010	0.067	0.006	0.075	0.003
291 - 300	NRDLVPDWLF	0.022	0.027	0.034	0.032	0.014	0.011	0.016	0.013	0.020	0.024	0.014	0.019	0.029	0.005	0.013	0.002	0.019	0.004
301 - 308	VAFNWLGY	0.739	0.737	0.798	0.695	0.687	0.539	0.701	0.591	0.676	0.544	0.589	0.577	0.742	0.037	0.630	0.067	0.596	0.049
304 - 308	NWLGY	0.008	0.008	0.009	0.011	0.005	0.005	0.005	0.005	0.005	0.006	0.005	0.005	0.009	0.001	0.005	0.000	0.005	0.000
309 - 318	ANSAMNPIIY	0.161	0.169	0.175	0.186	0.154	0.177	0.183	0.170	0.213	0.206	0.226	0.207	0.173	0.009	0.171	0.011	0.213	0.008
319 - 328	CRSPDFRKAF	0.034	0.038	0.038	0.034	0.019	0.024	0.025	0.022	0.023	0.027	0.026	0.023	0.036	0.002	0.023	0.002	0.025	0.002
333 - 342	AFPRKADRRL	1.000	1.000	1.000	1.000	1.000	1.000	1.000	1.000	1.000	1.000	1.000	1.000	1.000	0.000	1.000	0.000	1.000	0.000
333 - 349	AFPRKADRRLAAAENLY	1.000	1.000	1.000	1.000	1.000	1.000	1.000	1.000	1.000	1.000	1.000	1.000	1.000	0.000	1.000	0.000	1.000	0.000
343 - 349	AAAENLY	0.844	0.852	0.837	0.849	0.861	0.845	0.826	0.859	0.847	0.812	0.832	0.830	0.846	0.006	0.848	0.014	0.830	0.012

Table S5: Fractional modification values of tryptic β1AR peptides in the absence and presence of carazolol and carazolol + Nb60.

		Control				+ Carazolol				+ Carazolol + Nb60				Control		+ Carazolol		+ Carazolol + Nb60	
Peptide No.	Peptide	1	2	3	4	1	2	3	4	1	2	3	4	Mean	St.D	Mean	St.D	Mean	St.D
1 - 18	DYKDDDDASAWSHPQFEK	0.119	0.072	0.132	0.128	0.089	0.112	0.126	0.112	0.144	0.135	0.108	0.091	0.113	0.024	0.110	0.013	0.119	0.021
1 - 38	DYKDDDDASAWSHPQFEKGGGSGGGSGGSAWSHPQFEK	1.000	1.000	1.000	1.000	1.000	1.000	1.000	1.000	1.000	1.000	1.000	1.000	1.000	0.000	1.000	0.000	1.000	0.000
19 - 38	GGGSGGGSGGSAWSHPQFEK	0.094	0.120	0.105	0.096	0.111	0.072	0.101	0.079	0.098	0.096	0.071	0.077	0.104	0.010	0.091	0.016	0.085	0.012
143 - 151	YLAITSPFR	0.173	0.236	0.157	0.229	0.097	0.247	0.240	0.302	0.322	0.324	0.324	0.339	0.199	0.034	0.221	0.076	0.328	0.007
152 - 158	YQSLMTR	1.000	1.000	1.000	1.000	1.000	1.000	1.000	1.000	1.000	1.000	1.000	1.000	1.000	0.000	1.000	0.000	1.000	0.000
152 - 160	YQSLMTRAR	1.000	1.000	1.000	1.000	1.000	1.000	1.000	1.000	1.000	1.000	1.000	1.000	1.000	0.000	1.000	0.000	1.000	0.000
187 - 194	DEDPQALK	1.000	1.000	1.000	1.000	1.000	1.000	1.000	1.000	1.000	1.000	1.000	1.000	1.000	0.000	1.000	0.000	1.000	0.000
187 - 208	DEDPQALKCYQDPGCCDFVTNR	1.000	1.000	1.000	1.000	1.000	1.000	1.000	1.000	1.000	1.000	1.000	1.000	1.000	0.000	1.000	0.000	1.000	0.000
195 - 208	CYQDPGCCDFVTNR	0.015	0.009	0.020	0.018	0.012	0.007	0.010	0.007	0.009	0.009	0.007	0.007	0.016	0.004	0.009	0.002	0.008	0.001
252 - 259	TSRVMAMK	1.000	1.000	1.000	1.000	1.000	1.000	1.000	1.000	1.000	1.000	1.000	1.000	1.000	0.000	1.000	0.000	1.000	0.000
255 - 259	VMAMK	1.000	1.000	1.000	1.000	1.000	1.000	1.000	1.000	1.000	1.000	1.000	1.000	1.000	0.000	1.000	0.000	1.000	0.000
255 - 262	VMAMKEHK	1.000	1.000	1.000	1.000	1.000	1.000	1.000	1.000	1.000	1.000	1.000	1.000	1.000	0.000	1.000	0.000	1.000	0.000
321 - 325	SPDFR	1.000	1.000	1.000	1.000	1.000	1.000	1.000	1.000	1.000	1.000	1.000	1.000	1.000	0.000	1.000	0.000	1.000	0.000
321 - 326	SPDFRK	1.000	1.000	1.000	1.000	1.000	1.000	1.000	1.000	1.000	1.000	1.000	1.000	1.000	0.000	1.000	0.000	1.000	0.000
327 - 336	AFKRLLAFPR	1.000	1.000	1.000	1.000	1.000	1.000	1.000	1.000	1.000	1.000	1.000	1.000	1.000	0.000	1.000	0.000	1.000	0.000
330 - 336	RLLAFPR	0.665	0.713	0.620	0.611	0.616	0.622	0.587	0.648	0.587	0.577	0.553	0.565	0.652	0.041	0.618	0.022	0.571	0.013
331 - 336	LLAFPR	0.052	0.033	0.070	0.059	0.117	0.051	0.058	0.059	0.056	0.055	0.043	0.046	0.053	0.013	0.071	0.027	0.050	0.005
331 - 337	LLAFPRK	1.000	1.000	0.760	1.000	1.000	1.000	1.000	1.000	1.000	1.000	1.000	1.000	0.940	0.104	1.000	0.000	1.000	0.000
342 - 362	LAAAENLYFQGHHHHHHHHHH	0.451	0.450	0.338	0.349	0.316	0.374	0.354	0.374	0.323	0.326	0.341	0.308	0.397	0.054	0.355	0.024	0.325	0.012

Table S6: Fractional modification values of tryptic Nb60 peptides in the absence and presence of β1AR and β1AR + carazolol.

		Control				+ B1AR				+ B1AR +Carazolol				Control		+ B1AR		+ B1AR +Carazolol	
Peptide No	Peptide	1	2	3	4	1	2	3	4	1	2	3	4	Mean	St.D	Mean	St.D	Mean	St.D
1 - 21	MGQVQLQESGGGLVQAGGSLR	0.001	0.002	0.001	0.010	0.000	0.001	0.000	0.000	0.000	0.000	0.000	0.000	0.003	0.004	0.000	0.000	0.000	0.000
22 - 40	LSCAASGSIFSLNDMGWYR	0.000	0.000	0.000	0.003	0.000	0.000	0.000	0.000	0.000	0.000	0.000	0.000	0.001	0.001	0.000	0.000	0.000	0.000
46 - 60	LRELVAAITSGGSTK	0.046	0.061	0.058	0.035	0.049	0.030	0.047	0.049	0.031	0.047	0.074	0.058	0.050	0.011	0.044	0.008	0.053	0.016
48 - 60	ELVAAITSGGSTK	0.048	0.052	0.045	0.042	0.014	0.018	0.014	0.014	0.015	0.014	0.010	0.015	0.047	0.004	0.015	0.002	0.013	0.002
48 - 66	ELVAAITSGGSTKYADSVK	0.691	0.704	0.758	0.732	0.842	0.865	0.846	0.846	0.818	0.854	0.819	0.846	0.721	0.026	0.850	0.009	0.834	0.016
78 - 88	NTVYLQMNSLK	0.001	0.001	0.001	0.001	0.000	0.000	0.001	0.001	0.000	0.001	0.000	0.000	0.001	0.000	0.000	0.000	0.000	0.000
89 - 100	AEDTAVYYCNAK	0.068	0.052	0.054	0.074	0.020	0.022	0.030	0.030	0.024	0.033	0.032	0.019	0.062	0.009	0.025	0.004	0.027	0.006
101 - 129	VAGTFSIYDYWGQGTQVTVSSLEHHHHHH	0.000	0.000	0.000	0.000	0.000	0.000	0.000	0.000	0.000	0.000	0.000	0.000	0.000	0.000	0.000	0.000	0.000	0.000

Table S7: Raw data file descriptions

File Name	Description
JRL_101122_B1AR_C(1,2,3,4)	B1AR -isoprenaline, chymotryptic digest
JRL_101122_B1AR_ISO_(1,2,3,4)	B1AR +isoprenaline, chymotryptic digest
JRL_101122_B1AR_NB80_(1,2,3,4)	B1AR +isoprenaline +Nb80, chymotryptic digest
JRL_131222_B1AR_TRYP_C(1,2,3,4)	B1AR -isoprenaline, tryptic digest
JRL_131222_B1AR_TRYP_ISO_(1,2,3,4)	B1AR +isoprenaline, tryptic digest
JRL_131222_B1AR_TRYP_NB80_(1,2,3,4)	B1AR +isoprenaline +Nb80, tryptic digest
JRL_210323_B1AR_CHYM_C(1,2,3,4)	B1AR -carazolol, chymotryptic digest
JRL_210323_B1AR_CHYM_CARA_(1,2,3,4)	B1AR +carazolol, chymotryptic digest

JRL_210323_B1AR_CHYM_NB60_(1,2,3,4)	B1AR +carazolol +Nb60, chymotryptic digest
JRL_020323_B1AR_DMSO_C(1,2,3,4)	B1AR -carazolol, tryptic digest
JRL_020323_B1AR_CARA_(1,2,3,4)	B1AR +carazolol, tryptic digest
JRL_020323_B1AR_NB60_(1,2,3,4)	B1AR +carazolol +Nb60, tryptic digest
JRL_050423_NB80_C(1,2,3,4)	NB80 -B1AR, tryptic digest
JRL_050423_NB80_B1AR_(1,2,3,4)	NB80 +B1AR, tryptic digest
JRL_050423_NB80_B1AR_ISO_(1,2,3,4)	NB80 +B1AR +isoprenaline, tryptic digest
JRL_050423_NB60_C(1,2,3,4)	NB60 -B1AR, tryptic digest
JRL_050423_NB60_B1AR_(1,2,3,4)	NB60 +B1AR, tryptic digest
JRL_050423_NB60_B1AR_CARA_(1,2,3,4)	NB60 +B1AR +carazolol, tryptic digest
JRL_161122_B1AR_C(1,2,3,4)_MSMS_1; JRL_11222_B1AR_C(1,2,3,4)_MSMS_3	B1AR, -isoprenaline, chymotryptic digest, targeted MSMS
JRL_161122_B1AR_I(1,2,3,4)_MSMS_1; JRL_11222_B1AR_I(1,2,3,4)_MSMS_3	B1AR +isoprenaline, chymotryptic digest, targeted MSMS
JRL_161122_B1AR_NB(1,2,3,4)_MSMS_1; JRL_11222_B1AR_NB(1,2,3,4)_MSMS_3	B1AR +isoprenaline + Nb80, chymotryptic digest, targeted MSMS
JRL_191222_B1AR_TRYP_C(1,2,3,4)_MSMS	B1AR, -isoprenaline, tryptic digest, targeted MSMS
JRL_191222_B1AR_TRYP_ISO_(1,2,3,4)_MSMS	B1AR, +isoprenaline, tryptic digest, targeted MSMS
JRL_191222_B1AR_TRYP_NB80_(1,2,3,4)_MSMS	B1AR, +isoprenaline +Nb80, tryptic digest, targeted MSMS
JRL_120423_B1AR_CHYM_DMSO_(1,2,3,4)_MSMS_4	B1AR -carazolol, chymotryptic digest, targeted MSMS
JRL_120423_B1AR_CHYM_CARA_(1,2,3,4)_MSMS_4	B1AR +carazolol, chymotryptic digest, targeted MSMS
JRL_120423_B1AR_CHYM_NB60_(1,2,3,4)_MSMS_4	B1AR +carazolol +Nb60, chymotryptic digest, targeted MSMS
JRL_12323_B1AR_DMSO_CHYM_TRYP_MSMS_C(1,2,3,4)	B1AR -carazolol, tryptic digest, targeted MSMS
JRL_12323_B1AR_CHYM_TRYP_MSMS_CARA_(1,2,3,4)	B1AR +carazolol, tryptic digest, targeted MSMS
JRL_12323_B1AR _CHYM_TRYP_MSMS_NB60_(1,2,3,4)	B1AR +carazolol +Nb60, tryptic digest, targeted MSMS
JRL_130423_NB80_B1AR_C(1,2,3,4)_MSMS_1	Nb80 -B1AR, tryptic digest, targeted MSMS
JRL_130423_NB80_B1AR_(1,2,3,4)_MSMS_1	Nb80 +B1AR, tryptic digest, targeted MSMS
JRL_130423_NB80_B1AR_CARA_(1,2,3,4)_MSMS_1	Nb80 +B1AR +isoprenaline, tryptic digest, targeted MSMS
JRL_200323_NB60_B1AR_(1,2,3,4)_MSMS_1	Nb60 -B1AR, tryptic digest, targeted MSMS
JRL_200323_NB60_B1AR _(1,2,3,4)_MSMS_1	Nb60 +B1AR, tryptic digest, targeted MSMS
JRL_200323_NB60_CARA_(1,2,3,4)_MSMS_1	Nb60 +B1AR +carazolol, tryptic digest, targeted MSMS
